# Investigation of factors related to the behavior of reporting clinical errors in nurses working in educational and medical centers in Rasht city, Iran

**DOI:** 10.1186/s12912-022-01134-3

**Published:** 2022-12-08

**Authors:** Somayeh Abry, Fardin Mehrabian, Saeed Omidi, Mahmood Karimy, Parisa Kasmaei, Katayoun Haryalchi

**Affiliations:** 1 Department of Health Education and Promotion, School of Health, Guilan University of Medical Sciences, Rasht, Iran; 2 Department of Health Education and Promotion, Research Center of Health and Environment, School of Health, Guilan University of Medical Sciences, Rasht, Iran; 3 Guilan University of Medical Sciences, Rasht, Iran; 4 Department of Public Health, Social Determinants of Health Research Center, Saveh University of Medical Sciences, Saveh, Iran; 5 Department of Health Education and Promotion, Research Center of Health and Environment, School of Health, Guilan University of Medical Sciences, Rasht, Iran; 6 Department of Obstetrics & Gynecology, School of Medicine, Reproductive Health Research CenterAlzahra HospitalGuilan University of Medical Science, Rasht, Iran

**Keywords:** Clinical errors, Medical errors, Theory of planned behavior, Nurse

## Abstract

**Background:**

Report of medical error is one of the effective components in the quality of healthcare services. A significant part of medical errors can be prevented by acting appropriately. The theory of planned behavior offers a framework in which the nurse intention to perform the behavior of error reporting is investigated. This study was conducted to determine the factors related to the behavior of reporting clinical errors in nurses working in educational and medical centers in Rasht based on the theory of planned behavior in 2020.

**Methods:**

In this descriptive-analytical study, 326 nurses in all medical centers in Rasht were selected by the multi-stage random sampling method. Data collection tool was a valid and reliable questionnaire based on the theory of planned behavior. Data analysis was conducted using the SPSS software, analysis of variance, correlation, and linear regression.

**Results:**

39% of nurses reported that they had reported a medical error, and the average number of error reports per nurse during the last 3 months was 1.42 errors. The predictive power of the theory of behavioral intention was 47%, and predictive constructs were attitude (B = .43), perceived behavioral control (B = .33), and subjective norm (B = .04) using linear regression. The predictive power of the theory for nurses' behavior was 3.1%. None of the demographic variables played a role in predicting the behavior of nurses' reporting clinical error, and no behavioral intention predicted the behavior of nurses' reporting clinical errors.

**Conclusion:**

The theory of planned behavior expresses the factors affecting the behavior intention of nurses' reporting clinical errors satisfactorily. However, it was an inappropriate theory in behavior prediction. It appears that factors, such as fear of consequences of error reporting, social pressures by colleagues and officials, and lack of knowledge and skills required to identify medical errors, are the barriers to conversion of intention to the behavior of reporting clinical errors. It is necessary to provide the ground to increase nurses' report of clinical errors by acting appropriately.

**Supplementary Information:**

The online version contains supplementary material available at 10.1186/s12912-022-01134-3.

## Background

### Reporting of medical error

Medical errors are a serious public health problem and a leading cause of death in the world. All providers know that this problem poses a substantial threat to patient safety [1, 2]. Medical errors can occur at any stage of patient care, even during prevention [3]. Therefore, patient care and safety standards are one of the main indicators of health quality and nursing services [4]. Clinical activity is potentially dangerous and inherently unsafe [5]. Many people are unintentionally damaged by treatments in the process of curing [6]. Studies indicate that errors are not prominently reported in hospitals [7]. Consequently, numerous opportunities for improving quality and safety in hospitals are lost due to unreported errors [8]. In other words, timely report of clinical errors can help to prevent the recurrence of similar errors in the future and shorten the period of hospitalization [9, 10]. One out of 10 patients worldwide suffers from clinical errors. In the eastern Mediterranean, the damage rate is 2% to 18%. One of the largest studies in the region indicated that 14% of patients had permanent disability and 30% of patients died from clinical errors [11]. In this region, each adverse clinical event adds an average of 9.1 days to the number of hospitalization days. An estimation of the economic burden for low- and middle-income countries demonstrates that the average cost of adverse clinical events is $7295 million [11].

Medical errors are defined as unintentional consequences of care, which may lead to the patient’s physical damage [12]. Medical errors will cause direct and indirect complications [3]. Threatening the patient's life and increasing costs are among the direct complications of medical errors. Among its indirect complications are nurses' occupational damage and patients' reduced confidence in the system and its function [13]. Error reporting offers real documentations of errors and quasi-errors and provides healthcare providers with the knowledge needed to improve the safety and quality of the care delivered to patients [14]. In Iran, although there are no written statistics on the amount and type of medical errors, experts speculate that this rate is very high according to the number of complaints from doctors and nurses to the medical system and courts [3, 15]. Recently, a systematic review study in 9 hospitals in Iran has shown that the prevalence rate of medical errors ranged from 0.06% to 42% [16]. The difference in the tools used to check the prevalence of error reporting can be one of the reasons for this difference, so that the error screening method using patients' medical records is more accurate than the voluntary error reporting form [16]. Medical error reporting is one of the important components in the quality of healthcare services, so that a significant part of medical errors can be prevented [17].

### Theoretical framework

The theoretical framework of the present study is based on the theory of planned behavior (TPB). TPB has been used more than other theories of social cognition in the prediction of human behavior [18, 19]. TPB is based on the hypothesis that a personal behavior intention to perform a certain behavior predicts the actual behavior. Three main structures, namely behavioral attitudes, subjective norms, and perceived behavioral control influence this intention. Behavioral attitudes are personal attitudes toward a particular behavior. Subjective norms are an individual’s perception of family and social pressures to perform an activity. Perceived behavioral control is an individual’s perception of the difficulty of engaging in behavior. Change in a structure can cause a change in behavioral intent [20]. Review of the theories and models indicates that the theory of planned behavior is the most complete and appropriate theory to study behavior, since human behavior is a reflection of various factors and the knowledge of this causal network to influence factors affecting behavior; this is one of the most important points that behavioral sciences have pursued for many years [21]. This theory has been successfully used to predict patients and healthcare professionals’ intentions and behaviors. This theory is able to explain approximately 40% of the relationship between intention and health behavior. Consequently, it is claimed that this index has the potential to develop behavior change interventions, and this theory could be applied in the health field [22]. TPB has been largely applied to the fields of social psychology and health psychology to analyze the factors determining the adoption of different risk behaviors with prevention and health promotion purposes. However, this theory could be applied to the health field [23]. TPB offers a framework in which the nurse intention to perform the behavior of error reporting is investigated. Previous studies focused more on attitude and barriers of medical errors reporting, and few studies have been conducted on error reporting based on behavioral patterns [24–27]. This study was conducted to determine the factors related to the behavior of reporting medical errors in nurses working in medical centers in Rasht based on the theory of planned behavior. Based on the purpose, we attempted to answer these questions: What is the average number of error reports per nurse over the past 3 months? What is the predictability of each of the TPB structures in the behavioral intention of reporting the clinical errors of the studied nurses? What is the predictability of each of the TPB structures in the in the behavior of reporting the clinical errors of the studied nurses?

## Methods

### Study design and sampling

This descriptive-analytical study was conducted on 336 nurses employed in 8 public hospitals in Rasht from July to September 2020. According to the study by Seyedin et al. [25], and considering the type 1 error 5% and d equal to 0.15, 336 people were selected using the multi-stage random sampling method. First, all public hospitals in Rasht, which included 8 hospitals, were selected and investigated by the census method. Then, based on the list obtained from all nurses with at least one year of work experience in each of the medical centers by the researcher, 42 nurses from each hospital were selected by systematic random sampling and entered into the study. Inclusion criteria included at least one year of work experience [25] in medical centers in Rasht.

### Measures

The primary tool used was derived from a valid and reliable questionnaire developed by Seyedin et al. [25]. Owing to relocation of conducting research, the reliability of the tool was evaluated. For this purpose, 22 nurses completed the questionnaire. Internal correlation based on the Cronbach's alpha was obtained for each of the constructs (Table 2) and 0.72 for the whole tool. This tool, which is developed based on the theory of planned behavior (40 questions), includes: 1. Demographic characteristics (Table 1). This section contained items on gender, job experience, ward, job rank, age and number of patients covered by the nurse in a work shift. 2. Structures of the theory of planned behavior were included in the constructs derived from 33 items. Table 2 presents the number of questions and the range of values that can be obtained for this part. The answers to questions were in the form of 7 Likerts, and each question had a score from 1 to 7.

**Table 1:** Distribution of some demographic characteristics of the studied nurses

Demographic characteristics		N	Percent
Gender	Female	306	93.9
	Male	20	6.1
Work experience	Under 10 years	139	42.6
	10 to 20 years	167	51.2
	Over 20 years	20	6.1
Ward	Emergency	38	11.7
	Surgery	69	21.2
	Intensive Care Unit	71	21.8
	Children	10	3.1
	Internal	37	11.3
	Surgery room	11	3.4
	Other*	90	27.6
Job rank	Nurse	307	94.2
	Supervisor	19	5.8
Age	Under 30 years old	82	25.2
	30- 40 years old	169	51.8
	Over 40 years old	75	23
Number of patients covered	Less than 10	260	80.2
	10 to 30	56	17.3
	More than 30	8	2.5

^*^Obstetrics, gynecology, infectious disease, clinic, transplant, lung, skin, respiratory isolation, poisoning, methadone, neurology, radiotherapy, crusher

**Table 2:** Description of the structures of TPB in the studied nurses

Average percentage of the score obtained	Range of scores obtained	Range of scores that can be obtained	Standard deviation	Mean	Cronbach's alpha (α)	Number of questions	Variable
79	7–1.36	7–1	1.12	5.53	.652	10	Attitude
70.4	7–1	7–1	1.17	4.93	.736	10	Subjective norm
66/7	6/89–2.56	7–1	0.58	4.67	.770	9	Perceived behavior control
73	7–2	7–1	1.42	5.11	.665	4	Intention

There was a score that Option 1 indicates the minimum willingness, and Option 7 shows the maximum willingness and intention of the respondent to increase the items of medical error reporting.

Attitude: “If my patient is exposed to a medical error, the treatment period will be longer” and “If I report my medical error, I will improve the quality of medical care for future patients” are example items to measure this structure, which had 10 questions with a minimum score of 14 and a maximum score of 98 (Each of questions 9 and 10 had three parts).

Subjective norms: “Currently, most of my colleagues who are nurses have increased the error reporting to the authorities” and “I feel I am under (social) pressure to reduce the rate of reporting errors to high-ranking officials” are example items to measure this structure, which had 10 questions with a minimum score of 10 and a maximum score of 70.

Perceived behavioral control: “The decision as to whether or not I will report an error in the next three months is entirely up to me” and “At the hospital where I work, I can easily express my views on medical emergencies” are example items to measure this structure, which had 9 questions with a minimum score of 9 and a maximum score of 63.

Behavioral intention: “I intend to increase the percentage of error reports to the authorities in the next three months” and “In the next three months, I want at least one occasion not to tell my superiors that a medical error has occurred. Because I am worried about its legal responsibility" are example items to measure this structure, which had 9 questions with a minimum score of 4 and a maximum score of 28.

The behavior section consisted of 3 questions. The first question was “Have the nurses ever had a clinical error that delayed treatment or caused discomfort to the patient?” The second question was related to "The frequency of clinical error in the last 3 months", and the third question was regarding "The observance of the behavior of reporting clinical error by an experienced colleague or supervisor who has talked to others about it”. In the first and third questions, “yes option” had 3 points, “no option” had 2 points and “I do not know option” had 1 point. In addition, the third part of the questionnaire included 4 descriptive questions evaluating the participants’ views about clinical error qualitatively. The inverse questions in this questionnaire were 9.1, 13, 30, 31 and 37.

### Statistical analysis

The collected data were analyzed using the SPSS 21 software. Frequency tables, central tendency indices, such as average and dispersion indices like standard deviation, were used to describe the data according to the type of variable (quantitative or qualitative). Parametric and non-parametric tests (Wilcoxon and Chi-square) were used to determine the relationship between demographic characteristics, behavioral intention, and behavior score according to the normality or non-normality of the data, depending on the variables. Correlation test was used to determine the association between theory and behavior constructs and behavioral intention. Linear regression analysis was used to investigate the prediction of each of the constructs of the theory of planned behavior in behavioral intention and behavior, as well as the prediction of behavioral intention in behavior. The predictive rate of the theory was determined by the R2 index. *P* < 0.05 was considered in the statistical analysis.

## Results

A total of 336 questionnaires were administered in this research, of which 10 were omitted (e.g., missing data); accordingly, the final analysis was conducted on 326 nurses. Based on the results, 93.9% were female, and 6.1% were male. Furthermore, 51% of the nurses participating in this project were 30 to 40 years old. The work experience of the nurses was 10 to 20 years (51.2%); most in-service wards were special wards (21.8%), surgery (21.2%) and others (27.6%). On average, each nurse was responsible for caring 8 patients in each shift (Table 1). The average number of the error reports per nurse during the last 3 months was 1.42 errors (standard deviation = 2.68), and 39% of nurses had medical errors that delayed treatment or caused discomfort to the patient.

Table 2 shows the average score of the studied constructs. The average score of attitude was higher than that of other constructs, so that the nurses gained 79% of the maximum achievable score of the attitude construct. The constructs of behavioral intention, subjective norms, and perceived behavioral control gained 73, 70.4 and 66.7% of achievable scores, respectively. In the analytical study of the constructs, it was found that, in the attitude construct, the items "Serious errors should be reported to the authorities" with a frequency of 66%, "Reporting error can be a suitable tool for learning from harmful events" with a frequency of 58.3%, and “I think reporting error to the authorities is the right thing to do” with a frequency of 57.7% had the greatest impact on predicting nurses' behavioral intention. In the perceived behavioral control construct, the items "I am sure I can increase the percentage of reporting error to the organizational officials that I serve" with a frequency of 32.8%, "In the next three months, whether or not I report an error is entirely up to me” with a frequency of 29.4%, and “In the hospital where I work, I can easily express my views on medical accidents" with a frequency of 27.7% had the greatest effect on the prediction of behavioral intention.

Table 3 reveals that the most important advantage of reporting error according to the participants was the reduction of the frequency of errors and the increase of the quality of treatment (60.4%), and the most important disadvantage was the fear of inappropriate treatment, punishment, and pessimism (42.6%). The most important facilitator of reporting error was the encouragement and appropriate treatment of the individual (28.2%), and the most important obstacle was the fear of inappropriate treatment (30.7%).

**Table 3:** Advantages, disadvantages, facilitators of or barriers to medical errors reporting in the studied nurses

Question	Response	Frequency	Percent
What are the benefits of medical errors reporting?	Reducing the frequency of medical errors and increasing the quality of treatment	196	60.4
	Promoting patient safety and satisfaction	76	23.4
	Conscience, God	20	6.17
	Avoiding more serious mistakes	32	9.87
	Total	324	100
What are the disadvantages of medical errors reporting?	Fear of being mistreated, punished and pessimistic	136	42.6
	Not following up the report and providing feedback by the authorities	65	20.3
	Time-consuming medical errors recording	59	18.4
	Excessive magnification of minor medical errors	59	18.4
	Total	319	100
What factors or conditions facilitate medical errors reporting?	Encouraging and treating the person appropriately	90	28.2
	Increasing facilities	56	17.5
	See reports of error by others	22	6.8
	Low workload and sufficient time	39	12.2
	Not mentioning the name of the reporter and the department	30	9.4
	Timely and fast medical errors reporting	26	8.1
	Following up the medical errors report and removing its causes by the authorities	33	10.3
	Having a sense of empathy with the patient and his family	23	7.2
	Total	319	100
What factors and conditions make medical errors reporting difficult or impossible?	Fear of ill-treatment and legal punishment	97	30.7
	Group medical errors	31	9.8
	Systematization of reporting	33	10.4
	Lack of time to report	52	16.5
	Disclosure of an individual’s name	31	9.8
	Lack of support and follow-up of the report by the authorities	39	12.3
	Not accepting your mistake	32	10.1
	Total	315	100

The investigation of the linear correlation coefficient between the studied variables indicated a weak relationship between behavior and subjective norm, as well as a strong association between behavioral intention and attitude. The relationship between behavioral intention and perceived behavioral control was strong, and it was moderate with subjective norm. The relationship between attitude and subjective norm, as well as attitude and perceived behavioral control was moderate, and the relationship between subjective norm and perceived behavioral control was high (Table 4).

**Table 4:** Correlation matrix of the research variables

Variable	Attitude	Subjective norm	Perceived behavioral control	Intention	Behavior
Attitude	1				
Subjective norm	0.443**	1			
Perceived behavioral control	0.414**	0.617**	1		
Intention	0.588**	0.468**	0.541**	1	
Behavior	0.055	0.108*	-0.003	0.060	1

^*^P < 0.05 ^**^P < 0.01

The results of examining the prediction of theoretical constructs to predict nurses' behavioral intention demonstrated that the regression model was able to predict 47% of changes of the nurses' behavioral intention, and only attitude and perceived behavioral control constructs predicted the nurses' behavioral intention. Entering attitude into the regression model alone predicted 36% of behavioral intention changes (Table 5).

**Table 5:** Predictors of the nurses' intention to report clinical errors (N = 326)

Variable	Unstandardized Coefficients		Standardized Coefficients Beta	T	Sig	95% C.I. for EXP (B)		Adjusted R square
	B	Std. Error				Lower	Upper	
Constant	-0.172	0.331		-0.520	0.604	-0.824	0.480	47%
Attitude	0.484	0.054	0.439	9.012	< 0.001	0.378	0.589	
Subjective norm	0.044	0.058	0.041	0.764	0.446	-0.070	0.159	
Perceived behavioral control	0.507	0.083	0.332	6.109	< 0.001	0.343	0.670	

The examination of the regression analysis regarding the constructs of the theory of planned behavior to predict the nurses' behavior of reporting clinical error indicated that only the subjective norm construct predicted the nurses’ behavior. The percentage of variance explaining the nurses' behavior of reporting clinical error by this regression model (adjusted coefficient of the determination model R2) was 2.4%. In other words, the obtained regression model predicted only 2.4% of the changes in nurses' behavior of reporting error (Table 6).

**Table 6:** Predictors of the nurses' behavior to report clinical medical errors (*N* = 326)

Variable	B	Std. Error	Coefficients Wald	df	Sig	EXP (B)	95% C.I. for EXP (B)		R square
							Lower	Upper	
Constant	-1.3	0.83	2.452	1	0.117	0.273			2.8
Attitude	0.033	0.15	0.047	1	0.828	1.033	0.769	1.388	
Subjective norm	0.363	0.147	6.097	1	0.014	1.438	1.078	1.919	
Perceived behavioral control	-0.347	0.215	2.612	1	0.106	0.707	0.464	1.077	
Intention	0.089	0.141	0.397	1	0.528	1.093	0.829	1.44	

The results of examining the prediction of demographic variables demonstrated that only the demographic variable of organizational position (*p* = 0.048) predicted the nurses' behavioral intention, and none of the demographic and contextual variables played a role in predicting the nurses' behavior of reporting clinical error. Behavioral intention did not predict the nurses' behavior of reporting clinical errors.

## Discussion

In this study, factors related to the nurses' behavior of reporting clinical errors in educational and medical centers based on TPB were investigated. The results revealed that 39% of the nurses reported medical errors, and the average number of reporting error for these nurses per nurse during the last 3 months was 1.42 errors. Previous studies in this field reported different results. For example, in one study conducted by Dashti et al. [28], 86.4% of nurses in 6 months had at least one reporting error,. Moreover, in one study conducted by Raeisi et al. [29], 19.61% of nurses in 6 months, in one study conducted by Park and Kim [30], 27.9% of nurses in 6 months, and in one study conducted by Therady [24], 68% of nurses in 12 months had at least one reporting error. In addition, the average error report in one study conducted by Dashti et al. [29] was 3.6 errors in 6 months, in one study conducted by Park and Kim [30], 2 errors in 12 months, and in one study conducted by Therady et al. [24], it was 1–2 errors in 12 months per nurse. Time of study and cultural and managerial differences are the reasons for these differences.

The findings of our study indicated that nurses' attitude toward reporting clinical errors was the strongest predictor of their intention to report clinical errors, being consistent with many studies in which attitude was the strongest predictor of behavioral intention [27, 31]. However, in one study that had a similar target population to ours, the perceived behavioral control construct was the strongest predictor of intent [25]. In one study conducted by Hung et al. [32] and Petrova et al. [33], nurses with a positive attitude exhibited a strong intention to report errors. Experts of behavioral sciences maintain that attitude is based on the outcomes of the individual experience of behavior or the observance of others’ behavior, and after experiencing a behavior, positive or negative attitudes toward the outcomes of reinforced behavior act as a motivation to exhibit or not to exhibit the behavior [34–36]. Therefore, according to the importance of reporting medical errors, it is possible to report more errors in hospitals by adopting strategies in order to provide appropriate feedback to reporting medical errors.

Another predictor of nurses’ intention for reporting clinical errors was the PBC (Perceived behavioral control (construct. This construct is one of the important behavioral factors, since higher PBC creates and increases a positive feeling toward the desired behavior and reduces the perceived barriers to exhibit it [19], confirming previous studies indicating that PBC is one of the most important predictors of behavioral intention. For instance, studies conducted by Lapkin et al. [37], and Natan et al. [27] demonstrated that PBC was a significant predictor of behavioral intention. Accordingly, if nurses acquire the appropriate skills and high self-confidence to report clinical errors and have sufficient information, opportunity, and time, it may be helpful in making their decision to report the errors.

There is a basic assumption that people exhibit behaviors to be encouraged by those around them, and the behavior of people is influenced by those around them. In our study, the subjective norm construct alone predicted the nurses’ behavior, and the regression model in the presence of theoretical construct predicted 2.4% of changes in the nurses' behavior of reporting error, which was negligible. The finding shows that the nurses' behavior of reporting clinical errors is to some extent influenced by the views of others; however, there was no significant relationship between the intention of reporting error and the construct of subjective norm. Previous researches also reported similar results. For example, studies by Hager et al. [38] and Lapkin et al. [37] revealed that subjective norm had a weak negative effect on behavioral intention. Another similar study by Seyedin et al. [25] demonstrated a significant weak relationship between subjective norm and behavioral intention.

In the present study, none of the demographic variables, as well as attitude and perceived behavioral control constructs was involved in predicting the nurses' behavior of reporting clinical error. The findings are consistent with some studies in the field of health-related behaviors in which there was no relationship between positive attitude and behavior [19, 39, 40]. In our study, no significant relationship was observed between intention and behavior, and regression analysis showed that intention did not predict the behavior. According to our findings, in one study conducted by Seyedin et al. [25], no significant relationship was observed between intention and behavior. One of the reasons for not converting intention to behavior may be explained by the relatively high perceived barriers facing nurses in reporting error. The barriers include concern about and fear of the consequences of reporting errors, social pressure, reporting error according to officials’ wishes and opinions, lack of proper support and feedback from officials, an inadequate management system, high workload, lack of time, and lack of knowledge and skills to identify medical error. The finding is consistent with studies conducted by Zarei et al. [41], Nasiri et al. [10], Hashemi et al. [42]. It is crucial that the knowledge and skills required to report clinical errors in appropriate training courses be provided to nursing staff, and the barriers to and pressures on them should be reduced by developing appropriate management systems. Nursing managers should also design and implement a non-punitive reporting culture with protective rules for reporter individuals in formulating their organizational policies, so that nurses can be safely encouraged to report errors.

### Limitations

- 1. The use of a questionnaire and data collection through self-reporting caused the problem of recall and utilitarianism, which was inevitable due to the nature of the questionnaire.
- 2. Difficult access to some staff owing to high workload as well as conservatism of some nurses in completing questionnaires were the other limitations of the study.
- 3. Dissatisfaction of some nurses and officials with not receiving feedback from the research projects and consequently having little desire to cooperate in this research were among the limitations.

In this regard, holding briefing sessions; conducting continuous follow-ups; emphasizing the need for people's cooperation to use the results of this study in relevant studies into the behavior of reporting clinical errors in order to improve and promote the health of patients; keeping information confidential, and completing the questionnaire without entering names were employed to reduce the above limitations.

## Conclusion

In general, it can be stated that TPB was able to satisfactorily express the factors affecting the nurses' behavior of reporting clinical errors in educational and medical centers in Rasht; however, it had limited predictive power in predicting the behavior. To achieve the goal of increased error reporting by nurses, obstacles and challenges ahead such as threats and fear of being reprimanded by the authorities should be removed. Establishment of organizations supporting nurses is one of the factors facilitating the process of reporting without fear, so that they should be supported by legal and insurance funds. It is conceivable that after creating the necessary infrastructure in this regard, the facilities for its implementation will be provided. The findings can be useful in designing interventions aimed at increasing the clinical errors reported by nurses. Moreover, the construct of attitude as the strongest predictor of behavioral intention can be the focus of educational interventions. It is necessary to change nurses' attitude toward reporting medical errors. Nurses' behavioral beliefs and evaluation of behavioral consequences should be clarified. In this respect, group work is potentially useful to discuss and refute beliefs preventing nurses from reporting errors. It is recommended that nurses be instructed on the importance of and obligation to reporting medical errors, with an emphasis on acing according to the safety rules.

## Supplementary Information

**Additional file 1.** 

**Additional file 2.**

## Data Availability

All data generated or analyzed during this study are included in this published article [and its supplementary information files].

## Abbreviations

TPB: Theory of planned behavior
