# Supplementary material for: Investigation of factors related to the behavior of reporting clinical errors in nurses working in educational and medical centers in Rasht city, Iran
Source: BMC Nurs. 2022 Dec 8;21:348. doi: 10.1186/s12912-022-01134-3 (PMC9733308; doi:10.1186/s12912-022-01134-3)
Supplement: Supplementary file 2 — Additional file 2. [file 12912_2022_1134_MOESM2_ESM.pdf]

Data generated or analyzed during this study

behavior1	patientcove	ward	workexperi	age	hospital	Jobrank	gender
3	11	7	2	1	5	1	1
2	7	7	2	1	6	1	1
1	14	1	2	1	8	1	1
1	6	2	2	1	1	1	2
1	10	2	2	1	1	1	2
3	7	2	2	1	1	1	2
1	6	2	3	1	1	1	2
3	9	2	2	1	1	1	2
3	4	2	2	1	2	1	2
2	3	2	2	1	2	1	2
3	6	2	2	1	2	1	2
1	6	7	1.5	1	2	1	2
2	8	4	2	1	3	1	2
2	9	4	2.5	1	3	1	2
2	8	5	3	1	3	1	2
1	6	2	2	1	3	1	2
2	6	5	3	1	3	1	2
2	6	2	4	1	3	1	2
2	20	2	2	1	3	1	2
2	8	2	2	1	4	1	2
3	8	1	4	1	4	1	2
3	50	1	4	1	4	1	2
1	50	1	2.5	1	4	1	2
3	15	2	3	1	4	1	2
3	15	2	3	1	4	1	2
2	10	2	4	1	4	1	2
2	2	2	3	1	4	2	2
2	33	7	2	1	5	1	2
2	35	7	3	1	5	1	2
2	10	7	4	1	5	1	2
3	12	7	2	1	5	1	2
1	8	7	4	1	5	1	2
2	8	5	2	1	5	1	2
3	9	3	2	1	6	1	2
3	9	3	2	1	6	1	2
2	4	6	3	1	6	1	2
3	12	5	3	1	6	1	2
1	10	7	2	1	6	1	2
3	11	7	3	1	6	1	2
2	12	7	3	1	6	1	2
2	8	7	2	1	6	1	2
2	10	5	6	1	6	1	2
1	10	5	3	1	6	1	2
1	11	7	14	1	7	1	2
2	3	3	3	1	7	1	2
3	3	3	9	1	7	1	2
1	3	1	5	1	7	1	2
3	6	1	2	1	7	1	2
1	6	1	2	1	7	1	2
3	10	1	2	1	7	1	2
2	8	7	2	1	7	1	2

Data generated or analyzed during this study

3	5	2	2	1	7	1	2
3	2	3	7	1	7	1	2
2	12	7	3	1	7	1	2
1	12	7	2	1	7	1	2
2	4	2	10	1	7	1	2
3	12	7	3	1	7	1	2
1	12	7	2	1	7	1	2
3	5	2	2	1	7	1	2
3	2	3	3	1	7	1	2
3	4	7	2	1	8	1	2
2	5	5	4	1	8	1	2
3	3	7	3	1	8	1	2
3	3	3	3	1	8	1	2
1	6	1	4	1	8	1	2
3	10	5	2.5	1	8	1	2
2	5	1	2	1	8	1	2
3	8	5	2	1	8	1	2
2	8	5	2	1	8	1	2
2	10	1	2.5	1	8	1	2
3	10	5	2	1	8	1	2
2	5	1	3	1	8	1	2
1	6	1	2	1	8	1	2
1	4	7	2	1	8	1	2
1	4	3	2.5	1	8	1	2
1	4	3	3	1	8	1	2
2	7	2	1.5	1	8	1	2
3	3	3	2	1	8	1	2
2	4	3	2	1	8	1	2
1	4	3	2	1	8	1	2
2	7	5	1.5	1	8	1	2
2	3	3	2	1	8	1	2
3	50	1	12	2	4	1	1
2	15	2	10	2	4	1	1
3	11	7	8	2	5	1	1
1	11	1	11	2	6	1	1
1	4	3	15	2	7	1	1
2	16	7	10	2	7	1	1
2	6	2	14	2	7	1	1
2	3	2	16	2	7	1	1
3	3	7	12	2	7	1	1
2	7	2	16	2	7	1	1
2	5	2	16	2	7	1	1
2	7	4	10	2	1	1	2
1	6	4	2	2	1	1	2
2	2	7	10	2	1	1	2
3	2	7	13	2	1	1	2
1	3	3	13	2	1	1	2
1	3	3	9	2	1	1	2
1	3	3	12	2	1	1	2
2	5	3	10	2	1	1	2
3	8	7	12	2	1	1	2
3	8	7	12	2	1	1	2
2	2	3	13	2	1	1	2

Data generated or analyzed during this study

1	2	2	10	2	1	1	2
3	5	2	10	2	2	1	2
1	5	2	10	2	2	1	2
3	5	2	6	2	2	1	2
3	2	3	11	2	2	1	2
3	2	3	14.5	2	2	1	2
2	1	3	10	2	2	1	2
1	2	3	12	2	2	1	2
1	2	3	12	2	2	1	2
2	2	3	12	2	2	1	2
2	2	3	12.5	2	2	1	2
3	2	3	8	2	2	1	2
3	5	2	15	2	2	1	2
3	4	2	15	2	2	1	2
3	3	2	15	2	2	1	2
3	3	2	10.5	2	2	1	2
2	4	2	10	2	2	1	2
3	10	2	10.5	2	2	1	2
2	8	4	13	2	3	1	2
2	30	7	17	2	3	1	2
2	2	3	6	2	3	1	2
2	2	3	8	2	3	1	2
2	6	4	10	2	3	1	2
2	4	7	18	2	3	2	2
3	2	3	11	2	3	1	2
3	2	3	14	2	3	1	2
3	10	4	12	2	3	1	2
3	10	5	8	2	3	1	2
3	10	5	9.5	2	3	1	2
3	5	2	10	2	3	1	2
1	4	2	12	2	3	1	2
2	6	5	13	2	3	1	2
2	6	2	12	2	3	1	2
2	2	3	12	2	4	1	2
3	50	1	10	2	4	1	2
3	3	3	13	2	4	1	2
2	2	3	9	2	4	1	2
3	10	2	14	2	4	1	2
1	7	2	13	2	4	1	2
1	7	2	14	2	4	1	2
2	10	2	7	2	4	1	2
2	10	2	5	2	4	1	2
2	15	2	12	2	4	1	2
3	10	7	9.5	2	5	1	2
2	12	7	19	2	5	1	2
3	10	7	10	2	5	1	2
1	15	7	5	2	5	1	2
2	10	7	15	2	5	1	2
1	10	7	7	2	5	1	2
2	15	7	12	2	5	1	2
2	10	7	12	2	5	1	2
2	20	7	11	2	5	1	2
2	15	7	15	2	5	1	2

Data generated or analyzed during this study

2	10	7	12	2	5	1	2
2	5	1	10	2	5	1	2
2	5	1	12	2	5	1	2
3	4	1	9	2	5	1	2
2	5	1	8	2	5	1	2
2	5	1	13	2	5	1	2
2	5	1	11	2	5	1	2
2	3	1	7	2	5	1	2
1	10	7	18	2	6	1	2
3	4	3	17	2	6	1	2
2	8	5	10	2	6	1	2
2	3	2	9	2	6	1	2
3	7	5	7	2	6	1	2
3	14	7	10	2	6	1	2
2	10	2	14	2	6	2	2
2	12	1	12	2	6	1	2
3	12	1	12	2	6	1	2
3	11	7	12	2	6	1	2
3	10	7	15	2	6	1	2
2	10	1	16.5	2	6	1	2
3	12	7	15	2	6	1	2
3	4.5	3	14	2	6	1	2
3	12	1	13	2	6	1	2
2	12	5	12	2	6	1	2
3	10	1	11	2	6	1	2
3	4	5	14	2	6	1	2
3	7	5	10	2	6	1	2
2	4	5	13	2	6	1	2
1	3	5	7	2	6	1	2
3	5	5	18	2	6	1	2
3	6	5	16	2	6	2	2
3	5	5	2	2	6	1	2
3	9	5	12	2	6	1	2
2	1	2	10	2	7	1	2
2	10	7	15	2	7	1	2
3	15	7	10	2	7	1	2
3	2	3	13	2	7	1	2
2	8	2	14	2	7	1	2
1	8	7	14	2	7	1	2
3	8	2	12	2	7	1	2
2	10	7	16	2	7	1	2
3	10	7	10	2	7	1	2
1	2	3	12	2	7	1	2
3	10	1	18	2	7	1	2
2	2	3	14	2	7	1	2
1	1	3	13	2	7	1	2
1	2	3	14	2	7	1	2
3	2	3	10	2	7	1	2
2	2	3	15	2	7	1	2
2	2	3	7	2	7	1	2
1	6	7	8	2	7	1	2
1	4	6	14	2	7	1	2
3	3	6	14	2	7	1	2

Data generated or analyzed during this study

2	10	7	16	2	7	1	2
2	13	7	8.5	2	7	1	2
1	10	7	12	2	7	1	2
2	5	1	11	2	7	1	2
3	3	1	11	2	7	1	2
2	3	3	8	2	7	1	2
3	12	2	11.5	2	7	1	2
1	12	2	9	2	7	1	2
2	5	2	15	2	7	2	2
3	12	2	12	2	7	1	2
3	17	7	12	2	7	1	2
3	4	2	14	2	7	2	2
2	10	2	12	2	7	1	2
1	10	2	13.5	2	7	1	2
3	15	7	2	2	7	1	2
3	3	7	14	2	7	2	2
3	10	7	11	2	7	1	2
1	10	7	12	2	7	1	2
3	30	7	13	2	7	1	2
3	25	7	16	2	7	1	2
1	6	7	19	2	7	2	2
3	8	2	12	2	7	1	2
2	2	3	14	2	7	1	2
3	12	2	11.5	2	7	1	2
2	5	2	15	2	7	2	2
2	10	7	16	2	7	1	2
2	2	3	15	2	7	1	2
1	12	2	9	2	7	1	2
2	4	3	7	2	8	1	2
1	1	6	11	2	8	1	2
2	5	1	14	2	8	1	2
1	6	1	10	2	8	1	2
1	5	5	4	2	8	1	2
3	4	3	10	2	8	1	2
1	1	6	10	2	8	1	2
1	1	6	14	2	8	1	2
3	7	1	12	2	8	1	2
1	4	3	12	2	8	1	2
2	4	3	17	2	8	1	2
3	4	3	13	2	8	1	2
3	4	3	12	2	8	1	2
3	10	7	18	3	5	1	1
2	5	3	29	3	6	1	1
1	4	7	20	3	6	1	1
2	4	3	27.5	3	6	1	1
3	6	7	24	3	7	1	1
3	12	2	20	3	7	2	1
2	20	1	12	3	1	1	2
2	5	1	17	3	1	1	2
3	6	7	22	3	1	1	2
1	3	3	11	3	1	1	2
3	5	7	20	3	1	1	2
2	10	7	14	3	1	1	2

Data generated or analyzed during this study

2	2	3	20	3	1	1	2
2	2	3	14	3	2	1	2
3	2	3	20	3	2	2	2
3	5	2	19	3	2	1	2
1	4	7	14	3	3	2	2
2	15	7	27	3	3	1	2
2	20	7	20	3	3	1	2
1	4	7	18	3	3	1	2
1	13	4	17	3	3	1	2
3	7	4	28	3	3	2	2
2	3	7	29	3	3	2	2
2	3	3	17	3	3	1	2
3	2	3	19	3	3	1	2
2	35	7	14	3	5	1	2
2	10	7	15	3	5	1	2
2	10	7	18	3	5	1	2
1	15	7	17	3	5	1	2
1	10	4	10	3	5	1	2
3	8	7	15	3	5	1	2
2	4	1	27	3	5	1	2
2	5	1	10	3	5	2	2
3	6	7	21	3	6	1	2
2	5	7	18	3	6	1	2
2	4	3	25	3	6	1	2
1	4	3	25	3	6	1	2
2	4	3	20	3	6	1	2
2	3	2	15	3	6	1	2
1	5	3	18	3	6	1	2
3	8	5	18	3	6	1	2
3	5	6	19	3	6	1	2
2	3	6	17	3	6	1	2
3	10	6	20	3	6	1	2
3	4	5	19	3	6	2	2
3	12	5	24	3	6	1	2
3	12	7	15	3	6	1	2
1	4	3	15	3	6	1	2
3	4	3	21	3	6	1	2
1	3	3	19	3	6	1	2
1	24	5	18	3	6	1	2
1	10	5	20	3	6	1	2
3	5	5	18	3	6	1	2
2	4	2	20	3	6	1	2
3	7	2	18	3	6	1	2
3	6	6	19	3	6	1	2
2	15	5	18	3	6	1	2
3	6	5	23	3	6	1	2
3	10	5	20	3	7	1	2
3	33	7	18	3	7	2	2
3	5	2	13	3	7	1	2
2	8	2	24.5	3	7	2	2
3	2	7	15	3	7	1	2
3	3	6	28	3	7	1	2
3	12	7	21	3	7	1	2

Data generated or analyzed during this study

2	12	7	18	3	7	1	2
2	13	7	20	3	7	1	2
2	8	7	20	3	7	2	2
3	10	5	24	3	7	1	2
3	3	3	16	3	7	1	2
1	3	7	19	3	8	1	2
2	6	2	24	3	8	1	2
2	7	2	19	3	8	1	2
3	4	3	12	3	8	1	2
2	3	3	25	3	8	1	2

Data generated or analyzed during this study

attitude52	attitude51	attitude4	attitude3	attitude2	attitude1	behavior3	behavior2
7	7	7	7	1	7	3	1
7	7	7	7	7	7	2	0
4	5	4	5	5	5	2	0
7	7	3	3	1	4	2	0
7	7	7	7	7	7	3	1
7	7	7	7	2	7	3	2
7	7	3	3	1	4	3	0
7	7	2	4	2	7	1	3
7	1	7	7	7	7	3	1
6	5	4	5	6	5	3	0
7	1	6	6	6	7	3	1
3	4	2	3	3	3	2	1
7	7	7	7	7	7	3	0
7	1	4	5	5	7	2	0
6	7	5	3	3	5	2	0
4	7	7	7	4	1	2	1
5	5	5	5	6	6	3	1
5	3	4	4	4	4	1	0
5	3	4	4	5	5	3	0
7	7	7	7	7	7	2	0
1	1	3	3	3	4	3	0
7	7	7	6	6	7	2	3
7	7	7	7	7	7	2	0
7	7	7	7	7	7	3	3
4	1	3	4	6	4	3	2
4	4	4	3	3	3	2	0
7	7	5	5	3	7	3	0
6	4	3	4	5	7	3	0
6	7	6	5	6	7	3	0
7	7	7	7	7	7	1	0
7	4	4	4	7	3	1	1
7	7	4	7	7	7	2	0
7	4	5	5	5	7	1	0
6	2	3	3	3	3	3	1
7	2	1	6	4	4	3	2
7	6	5	5	2	6	2	0
7	7	7	7	7	7	3	1
7	7	4	5	5	3	3	0
3	4	4	3	6	6	3	2
4	4	5	4	6	6	3	0
7	7	3	5	4	6	2	1
7	5	5	5	4	4	2	0
7	6	4	6	6	5	3	10
4	4	6	7	6	7	1	0
5	4	4	5	4	3	2	0
7	7	4	7	7	6	3	0
7	3	5	7	6	7	1	0
7	2	5	7	6	4	3	2
7	1	4	3	5	4	1	0
4	5	3	2	3	3	1	0
5	3	5	5	5	1	3	0

Data generated or analyzed during this study

7	7	1	7	2	7	1	1
7	7	4	6	5	7	3	1
7	7	7	7	7	7	1	0
4	4	6	7	7	4	3	0
6	2	6	6	5	5	2	0
3	1	6	2	3	4	1	2
4	4	6	7	7	4	3	0
7	7	1	7	2	7	1	1
6	3	4	4	5	3	2	3
7	7	3	5	6	5	3	1
7	7	6	7	7	7	3	0
6	7	4	6	6	5	3	7
6	3	4	4	5	3	2	3
7	7	5	6	6	6	1	0
7	6	3	5	4	4	3	5
6	5	4	5	7	6	3	0
7	7	7	7	7	7	3	1
4	3	6	7	7	7	3	0
1	7	3	5	4	4	2	0
7	6	3	5	4	4	3	5
7	7	7	7	7	7	3	0
2	5	3	3	2	2	1	1
7	4	7	7	3	7	1	2
6	7	7	7	5	7	3	1
1	3	5	5	6	7	3	0
7	7	7	7	7	7	1	2
7	7	5	7	5	7	3	5
7	7	7	7	7	7	2	0
7	7	4	7	6	5	3	8
7	2	6	6	6	6	1	0
5	3	5	5	4	6	3	0
7	3	5	6	1	5	3	4
4	4	3	3	3	3	2	0
7	7	7	7	1	7	3	1
4	4	4	4	4	4	1	0
5	3	3	3	2	2	1	0
5	4	3	5	3	4	3	0
4	4	5	4	4	4	2	4
5	2	7	6	7	6	2	1
1	1	5	5	7	7	2	5
5	2	7	6	7	6	2	1
5	2	7	6	7	6	2	1
7	7	6	5	6	5	2	0
6	3	6	4	5	3	1	1
7	7	5	7	6	6	2	0
7	7	7	3	4	3	2	2
7	7	5	6	7	6	3	0
7	7	7	6	5	6	1	1
6	6	6	4	5	6	1	1
6	6	6	6	6	6	3	0
7	7	7	7	7	7	2	1
4	4	3	3	3	3	2	2
1	1	1	1	1	1	3	0

Data generated or analyzed during this study

7	7	7	7	6	7	2	2
7	1	7	7	7	7	3	1
7	7	1	7	7	7	1	2
1	1	7	7	7	7	3	1
3	2	3	4	4	4	3	1
1	4	2	4	3	5	3	2
7	7	7	7	6	6	3	0
7	7	7	7	7	7	2	0
7	7	7	7	7	7	2	0
5	6	4	4	6	5	2	0
7	4	5	5	5	6	1	0
7	7	7	7	7	7	2	1
5	5	7	7	7	7	2	4
6	7	6	6	7	7	3	2
7	7	7	7	7	7	3	1
7	7	4	5	7	7	3	1
7	7	7	7	7	7	2	0
7	1	6	6	6	7	3	1
7	7	7	7	7	7	2	3
7	1	7	7	7	7	3	0
7	7	3	4	5	3	1	3
5	3	7	7	7	7	1	0
7	7	5	7	7	7	2	0
7	7	7	7	3	7	3	2
7	4	1	7	7	7	3	3
7	4	1	7	7	7	3	1
6	2	7	7	7	7	2	1
5	3	3	3	2	3	1	2
7	7	7	7	7	7	1	1
7	7	7	7	7	7	3	1
7	7	7	7	7	7	2	0
5	3	5	5	6	6	1	0
7	7	4	4	5	7	3	5
6	6	5	6	1	7	2	2
7	7	7	6	6	7	2	2
7	7	6	6	3	7	1	2
7	7	6	6	7	6	3	0
4	4	4	4	4	4	2	3
3	5	4	4	3	4	1	0
3	5	4	4	3	4	1	0
5	3	4	4	4	4	2	0
4	4	5	5	5	4	2	0
7	7	7	6	7	7	2	1
3	4	4	3	2	3	2	2
6	3	6	7	7	6	3	0
7	7	7	7	7	6	3	1
4	4	5	5	5	4	1	0
7	7	6	6	6	7	1	0
4	5	2	7	7	7	2	0
3	5	7	7	6	7	2	1
5	3	4	5	4	5	2	0
3	5	7	7	6	7	2	0
6	7	6	6	6	6	2	0

Data generated or analyzed during this study

7	7	3	7	7	7	2	0
7	1	7	7	6	7	2	0
7	7	7	7	7	7	2	0
7	7	7	7	7	7	2	5
7	1	7	7	6	7	1	0
7	1	7	7	6	7	2	0
7	4	4	5	7	6	2	0
7	1	7	7	6	7	2	0
7	1	0	5	3	5	1	0
4	4	4	5	4	4	2	1
6	2	6	6	6	6	3	0
6	6	7	6	5	6	2	3
7	7	5	5	5	5	3	1
7	1	7	7	7	7	3	0
7	7	7	7	7	7	2	0
6	7	6	6	5	7	1	0
6	6	5	4	4	6	2	2
4	7	1	4	2	5	3	0
4	1	3	6	5	7	3	1
7	7	7	7	3	7	3	0
7	7	7	7	6	7	1	1
5	6	7	7	6	6	3	5
7	7	7	7	7	7	3	4
1	7	6	6	5	5	3	0
6	6	5	5	5	4	3	0
7	7	7	7	7	7	3	3
6	5	3	4	4	5	3	0
6	4	5	5	7	5	1	2
7	7	7	7	6	7	3	20
7	7	7	7	4	7	1	1
1	2	6	6	6	7	3	2
7	7	6	7	7	7	3	2
1	7	6	7	7	7	3	1
4	4	4	4	4	4	2	0
6	7	6	6	7	7	3	0
7	7	7	7	7	7	2	0
7	7	6	6	7	6	1	3
5	3	4	4	4	4	2	0
4	1	4	4	1	7	3	0
3	6	5	4	5	4	2	2
7	7	1	6	1	4	2	0
7	5	7	7	7	7	1	1
7	7	6	6	5	4	2	0
5	3	1	4	5	4	2	4
7	7	1	7	3	7	3	0
7	7	7	7	6	7	2	2
7	7	1	7	2	7	2	0
7	1	7	6	7	7	2	1
1	1	1	1	1	1	3	0
1	1	1	1	1	1	3	0
4	4	4	4	4	4	1	0
6	2	5	4	6	5	3	0
4	1	7	7	7	7	3	3

Data generated or analyzed during this study

4	5	3	7	6	6	1	0
6	4	4	6	6	5	2	0
7	7	3	4	4	4	1	0
7	4	7	7	7	7	2	0
7	7	7	7	7	7	1	4
7	7	7	7	7	7	2	0
6	7	4	5	5	5	3	1
4	4	5	5	5	5	3	2
7	7	7	7	7	7	3	0
6	2	6	6	6	6	3	1
6	3	6	6	6	6	3	2
1	1	7	7	7	7	3	2
7	7	3	7	4	7	3	0
4	7	7	7	7	7	3	0
7	7	7	7	7	4	3	3
1	1	5	5	7	7	2	5
4	4	7	7	7	7	1	0
5	5	5	5	6	6	1	0
1	2	6	6	1	7	1	10
1	2	6	6	1	7	2	10
4	1	7	7	7	7	1	0
3	6	5	4	5	4	2	2
7	7	1	7	3	7	3	0
6	7	4	5	5	5	3	0
7	7	7	7	7	7	3	0
4	5	3	7	6	6	1	0
4	7	1	1	1	1	3	0
4	4	5	5	5	5	3	2
7	7	3	2	6	6	2	0
6	6	6	6	4	7	3	0
7	7	4	4	2	7	3	2
7	7	7	7	6	5	3	1
4	4	4	4	4	4	1	0
7	7	7	7	7	7	3	1
6	6	6	6	4	7	3	1
6	6	6	6	4	7	3	0
7	7	1	7	5	7	2	4
7	7	7	7	7	7	2	3
7	7	3	2	6	6	2	0
7	5	4	4	7	7	1	1
2	2	3	7	4	6	3	1
5	5	4	4	4	3	2	0
7	7	3	6	2	6	2	0
7	3	6	7	5	6	1	2
7	7	4	5	3	3	2	0
5	2	1	5	3	7	1	10
7	7	4	7	7	7	2	10
7	7	7	7	7	7	2	0
7	7	4	4	4	7	2	0
7	7	7	7	4	7	3	0
5	5	5	5	4	4	1	0
7	7	7	7	7	7	3	2
6	2	7	5	5	5	3	0

Data generated or analyzed during this study

1	1	1	1	1	1	3	0
4	4	3	5	2	3	2	0
4	1	1	7	7	7	1	1
7	7	6	5	4	6	1	2
7	7	7	7	7	7	3	2
7	1	7	7	7	7	3	0
7	7	7	7	7	7	2	1
7	7	7	7	7	7	3	0
7	1	7	7	6	7	2	0
7	7	5	6	6	7	3	1
7	7	2	6	7	4	3	0
7	7	2	6	6	5	1	0
7	4	1	7	7	7	3	1
4	4	7	7	1	7	2	0
4	7	5	4	4	4	2	0
5	3	5	5	4	5	2	0
6	3	1	5	5	4	2	0
7	7	7	7	7	7	1	0
7	7	5	7	5	7	1	3
7	1	6	5	6	7	2	0
7	1	7	7	6	7	2	0
7	7	7	7	7	7	2	1
2	6	2	2	2	2	2	0
7	7	5	7	5	6	2	0
5	4	4	3	6	4	1	1
7	7	6	6	6	6	2	0
7	7	6	7	7	7	2	2
7	7	5	7	2	4	3	2
7	5	4	4	3	5	3	3
5	5	5	5	6	6	2	3
6	4	6	6	5	5	1	3
7	7	3	6	5	5	1	1
7	7	7	7	7	7	3	5
7	1	6	5	7	7	3	3
7	2	6	6	4	5	1	1
7	7	5	5	3	3	2	0
7	7	7	7	7	7	1	3
7	7	5	5	5	5	3	0
7	7	5	5	5	5	2	1
7	6	4	4	4	4	2	0
7	7	6	6	7	7	1	6
7	7	7	7	4	7	1	6
7	7	5	7	7	7	3	1
7	7	7	7	7	7	3	2
6	6	7	7	6	4	3	10
7	7	7	7	4	7	1	20
4	4	5	2	2	2	3	1
4	7	7	7	7	7	3	0
4	4	4	4	4	4	2	4
7	7	7	7	7	7	1	0
7	7	6	7	4	7	1	3
7	7	7	7	4	7	1	1
1	7	3	4	3	4	2	1

Data generated or analyzed during this study

6	4	6	5	5	5	2	0
7	7	7	7	7	7	3	0
7	7	6	6	7	7	3	1
7	7	7	7	4	7	1	1
7	7	7	7	4	7	1	3
7	7	4	3	3	1	1	0
7	7	7	7	7	6	2	20
7	7	7	7	7	7	3	10
7	4	7	7	1	7	3	1
7	7	3	7	6	6	2	5

Data generated or analyzed during this study

attitude10	attitude9	attitude8	attitude7	attitude63	attitude62	attitude61	attitude53
7	7	7	7	7	7	7	7
6	5	5	4	7	7	7	7
6	4	5	3	4	5	4	4
7	3	4	4	7	7	7	7
7	1	7	7	7	7	7	7
7	7	7	7	7	7	7	7
7	3	4	4	7	7	7	7
7	7	7	7	7	7	7	7
7	7	7	7	7	7	7	7
7	2	5	7	3	2	7	6
6	3	7	7	7	7	7	7
2	5	4	2	2	2	3	3
7	3	7	6	4	5	5	7
7	1	7	7	7	7	7	7
7	3	5	5	2	5	7	7
7	3	7	5	6	7	7	6
5	6	5	5	5	5	5	5
4	3	3	5	3	5	4	5
5	4	4	3	5	3	6	5
7	7	4	5	4	4	7	7
7	7	7	7	7	7	7	1
7	1	7	7	1	1	7	7
7	7	7	6	6	7	7	7
6	1	7	7	7	6	7	7
6	5	7	7	5	4	7	4
4	4	4	4	4	4	4	5
7	1	7	7	7	7	7	1
7	2	5	6	4	5	6	6
5	4	5	7	5	4	7	6
7	1	7	7	1	4	7	7
7	1	7	7	5	4	7	4
7	5	7	7	7	7	6	7
5	4	7	7	6	2	7	7
6	1	5	7	5	1	7	6
6	2	4	7	5	1	7	7
5	6	7	4	7	5	7	7
7	7	7	7	7	7	7	7
6	6	6	7	6	5	5	7
7	2	7	3	6	7	7	3
7	2	7	5	6	7	7	4
7	7	7	7	7	7	7	7
6	7	2	1	1	4	4	6
7	2	7	7	7	6	7	7
5	5	4	5	3	3	5	7
3	5	2	6	6	4	2	6
7	7	6	7	7	7	7	7
7	2	7	7	7	4	7	7
7	2	5	4	5	6	4	7
7	2	6	6	5	6	2	7
2	4	6	4	4	6	6	4
4	4	5	3	3	3	4	5

Data generated or analyzed during this study

7	1	6	6	7	5	7	7
6	5	6	5	6	4	5	7
7	7	7	7	7	7	7	7
6	1	5	7	7	3	7	4
4	3	5	6	6	6	5	7
3	5	3	3	3	3	3	4
6	1	5	7	7	3	7	4
7	1	6	6	7	5	7	7
3	4	5	3	4	5	3	6
6	2	6	7	7	7	7	7
7	5	7	3	6	7	7	7
6	5	5	7	1	2	7	1
3	4	5	3	4	5	3	6
6	4	7	7	7	7	7	7
5	7	7	7	4	6	7	7
4	7	6	7	7	7	7	5
7	7	7	7	7	7	7	7
7	1	6	7	7	7	7	4
5	5	3	4	2	4	2	2
5	7	7	7	4	6	7	7
7	3	7	7	4	3	7	7
1	7	1	1	1	1	1	3
4	4	7	3	4	1	7	7
6	4	5	6	6	6	7	7
7	7	6	5	7	6	7	1
7	1	7	7	7	7	7	7
7	1	6	6	7	2	7	7
3	4	3	2	2	3	4	7
5	4	6	6	6	6	6	6
6	2	6	6	6	6	6	7
4	3	5	5	6	5	6	3
7	1	7	5	1	7	7	7
4	4	4	4	4	4	4	4
7	7	7	7	7	7	7	7
4	4	4	5	7	5	7	4
2	6	2	3	3	1	1	5
6	5	6	4	5	4	5	4
4	4	4	4	4	4	4	4
6	2	6	5	5	6	6	6
7	7	7	7	2	2	3	1
6	2	6	5	5	6	6	6
6	2	6	5	5	6	6	6
7	1	7	7	7	7	7	7
4	4	3	4	5	4	6	7
7	7	4	6	5	1	4	4
7	4	5	4	7	3	4	7
7	2	6	6	3	5	7	7
6	2	7	7	4	4	7	7
6	1	6	6	6	6	7	7
7	1	7	7	7	2	7	6
1	2	7	7	6	5	5	7
3	4	3	4	3	3	3	4
1	3	1	1	1	1	4	1

Data generated or analyzed during this study

7	7	7	7	7	7	7	7
7	1	7	7	7	7	7	7
7	7	7	7	7	7	7	7
7	1	7	1	7	7	7	1
5	3	5	5	7	5	4	5
6	7	7	5	6	6	4	7
7	7	7	7	7	7	7	7
7	7	7	7	7	7	7	7
7	7	7	7	7	7	7	7
5	5	7	7	7	7	7	6
5	2	7	7	7	7	7	7
7	6	7	7	7	1	7	7
7	1	5	7	7	7	7	5
5	3	5	6	5	5	5	6
7	7	7	7	7	1	7	7
6	7	6	5	7	7	7	7
7	6	7	7	7	7	7	7
6	3	7	7	7	7	7	7
7	7	7	7	7	7	7	7
7	7	7	7	7	7	7	7
6	2	5	6	7	5	7	7
6	3	6	5	5	4	7	4
7	3	7	5	3	6	7	7
7	7	7	7	7	7	7	7
7	7	7	7	7	7	7	7
7	7	7	7	7	7	7	7
7	7	7	7	1	1	7	6
4	4	4	4	3	3	3	5
7	7	7	7	1	1	7	7
7	1	7	7	7	7	7	7
7	6	7	7	7	7	7	7
5	3	5	5	5	5	5	5
7	7	7	4	3	7	2	7
7	6	7	7	7	6	2	6
7	1	7	7	1	1	7	7
7	1	7	7	4	5	7	7
7	1	7	7	7	4	7	7
5	3	5	5	5	5	4	4
4	4	3	3	4	5	7	3
4	4	3	3	4	5	7	3
3	4	3	3	3	3	3	5
4	4	4	4	4	4	4	4
7	3	7	7	7	4	7	7
3	4	3	3	4	4	3	3
5	3	6	7	4	3	6	5
5	2	5	4	7	5	7	7
4	5	4	5	5	4	4	4
6	2	7	7	7	7	7	7
3	6	5	2	6	1	7	4
5	2	5	7	5	1	5	6
4	3	4	5	4	5	4	5
5	2	5	7	5	1	5	6
6	4	6	6	7	6	7	6

Data generated or analyzed during this study

7	7	7	7	7	7	7	7
7	1	7	1	7	7	7	7
7	1	7	7	7	7	7	7
7	1	7	7	7	7	7	7
7	1	7	1	7	7	7	7
7	1	7	1	7	7	7	7
6	2	5	7	4	4	7	7
7	1	7	1	7	7	7	7
4	7	7	7	5	5	7	7
4	6	5	3	4	3	1	3
6	2	6	6	6	6	6	6
7	1	6	7	6	6	6	7
7	7	7	7	7	7	7	4
7	1	7	7	7	7	7	7
7	7	7	7	7	1	7	7
6	3	7	7	7	7	6	6
6	2	6	6	6	6	6	6
7	1	5	4	7	7	7	4
7	7	7	7	7	7	7	4
6	7	7	7	7	5	2	7
7	7	7	7	7	7	7	7
5	6	5	7	5	5	6	7
7	7	7	7	7	7	7	7
5	5	6	6	7	5	7	1
5	6	5	6	2	5	7	6
7	7	7	7	7	7	7	7
6	6	6	7	4	6	6	7
4	4	7	7	5	5	5	6
7	1	7	7	7	7	7	7
7	7	7	7	7	7	7	7
6	5	7	5	5	6	7	7
7	4	4	4	1	1	1	5
7	4	4	4	1	1	1	1
5	5	3	3	3	4	4	4
6	6	7	7	7	5	7	6
7	1	7	7	7	7	7	7
6	6	7	7	7	7	7	7
4	4	4	4	4	4	4	5
7	1	7	7	7	7	7	4
6	3	2	6	7	7	7	3
2	2	2	6	1	7	2	7
7	2	6	5	7	7	7	7
7	7	6	7	7	7	7	7
7	1	7	7	1	1	2	5
7	4	7	7	7	7	7	7
7	7	7	7	6	7	7	7
5	1	7	6	7	5	7	7
6	1	7	7	6	6	7	7
1	7	1	1	1	1	4	1
5	5	6	4	4	3	6	1
7	1	7	7	7	7	7	4
6	7	7	6	7	7	7	6
7	7	7	7	7	7	7	4

Data generated or analyzed during this study

3	2	6	5	2	2	2	3
4	1	7	7	5	2	7	6
5	2	2	5	3	3	5	7
6	2	7	7	7	1	7	3
7	7	7	7	7	6	7	7
5	5	7	7	7	5	7	7
5	6	6	5	7	7	7	7
5	3	5	4	5	5	5	4
7	7	7	7	7	1	7	7
6	2	6	6	7	7	7	6
6	7	7	7	7	7	7	6
7	4	7	7	7	7	7	1
7	5	7	7	7	7	7	7
7	7	7	7	7	7	7	4
3	1	7	7	5	3	7	7
7	7	7	7	2	2	3	1
7	4	7	7	7	5	5	7
6	3	5	6	6	4	5	5
7	7	7	5	1	5	7	1
7	7	7	5	1	5	7	1
7	1	7	7	7	7	7	4
6	3	2	6	7	7	7	3
7	4	7	7	7	7	7	7
5	6	6	5	7	7	7	7
7	7	7	7	7	1	7	7
3	2	6	5	2	2	2	3
1	1	2	1	1	1	4	1
5	3	5	4	5	5	5	4
7	7	7	7	7	7	7	7
7	1	7	7	7	7	7	6
7	7	7	7	7	7	7	7
7	1	7	7	7	7	7	1
5	1	7	6	7	7	7	4
7	7	7	7	7	7	7	7
7	1	7	7	7	7	7	6
7	7	7	7	7	7	7	6
7	7	7	7	7	7	7	7
7	7	7	7	7	7	7	7
7	7	7	7	7	7	7	7
6	4	7	6	4	2	7	4
7	3	7	6	6	5	5	2
5	3	4	7	7	6	5	5
7	7	7	7	7	7	7	7
2	1	7	7	7	7	7	6
7	5	7	7	7	7	6	7
7	3	7	6	7	7	7	6
7	7	7	7	7	7	7	7
7	7	7	7	7	7	7	7
7	7	7	7	7	7	7	7
7	3	7	7	7	4	7	7
3	5	3	5	3	4	3	5
6	7	5	7	5	5	7	7
5	4	6	5	5	6	7	6

Data generated or analyzed during this study

1	3	2	1	1	1	4	1
5	5	1	1	5	5	6	5
7	1	5	7	1	1	1	7
6	7	7	7	2	4	7	7
7	7	7	7	7	7	7	7
7	7	7	7	7	7	7	7
7	7	7	7	7	7	7	7
7	1	7	7	7	7	7	7
6	3	7	7	7	6	7	7
7	7	7	7	7	7	7	7
7	3	7	7	7	3	7	7
5	5	6	5	5	2	5	7
7	7	7	7	7	7	7	7
5	4	4	4	4	4	4	7
4	4	4	5	4	5	4	4
5	3	4	5	4	5	4	5
6	3	7	7	3	3	3	6
7	7	7	7	7	7	7	7
7	7	7	7	7	7	7	7
3	6	6	7	6	2	6	7
7	1	7	1	7	7	7	7
7	1	7	7	7	7	7	7
2	6	2	2	3	2	2	2
7	7	7	7	7	7	7	7
6	7	6	4	7	6	6	7
7	7	7	7	7	7	6	7
7	5	7	7	7	7	6	7
5	2	5	3	1	2	7	7
7	7	7	7	5	2	7	7
5	3	7	7	7	2	6	5
6	6	7	6	6	6	6	6
7	6	5	7	5	6	7	7
7	7	7	7	7	4	7	7
7	1	7	7	7	7	7	7
7	6	7	7	6	7	6	6
7	3	7	7	7	7	7	7
7	7	7	7	7	1	1	7
7	7	7	7	7	7	7	7
6	2	7	7	7	7	7	7
7	7	4	6	5	5	5	7
7	6	7	7	7	2	2	7
7	7	7	7	7	3	7	7
7	7	7	7	7	1	1	7
7	1	7	7	7	7	7	7
7	2	7	7	5	4	7	5
7	7	7	7	7	7	7	7
2	5	4	4	7	7	1	4
7	1	6	3	7	7	7	4
4	3	4	3	4	5	4	4
7	7	7	5	7	7	7	7
7	7	6	6	7	7	7	7
7	7	7	7	7	7	7	7
5	1	7	7	4	5	5	1

Data generated or analyzed during this study

4	1	7	7	1	2	7	5
7	7	7	7	7	7	7	7
7	7	7	7	7	7	7	7
7	7	7	7	7	7	7	7
7	7	7	7	7	7	7	7
7	6	7	5	4	4	7	7
7	1	7	7	6	6	7	7
7	1	7	7	7	7	7	7
7	1	7	7	7	1	7	4
7	7	7	7	7	7	7	7

Data generated or analyzed during this study

subjective8	subjective7	subjective6	subjective5	subjective4	subjective3	subjective2	subjective1
7	7	7	7	7	7	7	7
5	3	5	3	5	4	2	4
4	3	4	4	5	6	5	2
6	6	6	6	4	4	1	3
7	7	7	7	7	7	7	7
7	7	7	3	7	7	2	5
7	7	1	4	1	4	1	3
7	7	7	2	4	4	4	2
7	7	7	7	7	7	7	7
2	2	1	4	3	3	3	3
1	1	5	7	7	7	7	4
2	2	3	5	2	2	2	3
6	4	1	7	7	7	7	5
5	5	5	5	4	5	5	5
6	4	6	5	6	6	1	5
7	4	5	5	7	5	7	5
3	2	5	5	5	5	5	5
4	5	5	5	4	3	5	3
4	3	3	5	4	4	5	5
1	1	1	7	1	1	3	5
7	7	7	7	5	7	7	5
6	7	7	7	4	5	5	1
4	3	1	4	1	4	4	4
7	7	7	5	6	6	7	6
4	3	4	4	2	4	6	6
4	4	3	3	4	3	4	4
5	4	1	7	3	7	6	3
6	4	5	4	5	5	4	1
4	4	4	6	5	5	5	5
7	7	7	7	7	7	7	7
5	4	1	3	2	5	2	6
3	5	6	5	2	5	7	5
4	4	4	5	4	6	7	6
4	4	5	6	3	3	5	3
5	5	2	6	4	3	5	1
6	6	3	2	6	4	4	3
7	7	7	7	7	7	7	7
3	4	6	4	3	3	4	3
6	2	4	6	4	7	7	6
5	3	5	3	3	7	7	6
6	7	7	7	4	7	7	1
4	5	4	7	5	6	7	4
6	6	7	7	4	6	6	4
5	3	4	5	6	4	3	5
3	3	3	6	4	5	3	3
2	1	1	7	3	7	6	5
7	4	7	6	5	6	7	6
6	7	6	5	5	5	5	6
5	3	5	6	6	6	7	7
3	2	5	5	5	5	2	2
4	4	2	6	2	4	5	4

Data generated or analyzed during this study

2	1	2	3	4	6	3	5
5	4	5	5	4	5	6	6
1	5	4	7	5	5	5	7
1	5	4	7	5	5	6	4
5	5	5	7	7	7	6	5
5	1	4	5	4	4	7	4
1	5	4	7	5	5	6	4
2	1	2	3	4	6	3	5
4	4	3	6	4	4	5	5
2	2	3	5	4	2	6	6
7	7	7	7	4	7	7	3
5	5	6	6	6	2	5	5
4	4	3	6	4	4	5	5
7	7	7	7	5	4	6	1
4	3	5	7	7	1	7	5
7	7	7	5	3	4	6	4
7	6	6	6	1	7	7	7
4	5	1	7	7	2	5	7
3	5	2	6	3	5	5	3
4	3	5	7	7	1	7	5
5	5	1	5	5	1	4	4
1	1	1	1	1	1	1	1
1	7	5	7	7	7	5	6
2	2	4	4	1	6	6	2
5	5	6	6	1	6	7	6
7	7	7	7	7	7	7	7
3	2	7	7	3	7	7	7
3	5	2	6	3	5	4	4
6	5	5	5	5	2	5	2
6	6	6	6	6	6	6	6
6	5	3	6	6	5	5	6
2	1	4	5	2	5	5	5
4	4	4	5	3	3	4	4
7	7	7	7	7	7	7	7
4	4	4	3	6	5	5	5
3	3	3	4	2	2	3	3
6	4	5	5	5	4	5	4
4	4	4	4	4	4	4	4
6	5	5	6	6	6	5	6
6	6	5	7	4	6	7	6
6	5	5	6	6	6	5	6
6	5	5	6	6	6	5	6
7	7	7	3	1	7	7	7
4	5	4	4	4	4	4	2
5	4	4	5	6	6	7	7
3	5	4	7	3	4	4	3
6	5	2	6	3	6	6	5
7	7	7	7	5	7	7	6
7	7	5	6	7	5	6	7
7	7	7	7	2	7	7	7
4	4	3	6	4	4	2	4
4	3	3	4	4	3	3	4
2	1	1	1	2	7	7	5

Data generated or analyzed during this study

5	4	4	5	6	6	7	7
7	7	7	7	7	7	7	7
1	3	1	7	1	7	7	7
7	7	7	7	7	7	7	7
6	6	6	7	5	5	4	3
6	7	7	6	3	5	6	7
7	7	7	7	5	7	7	7
1	1	1	7	1	4	6	4
1	1	1	7	1	4	6	4
5	5	5	6	6	6	7	4
6	5	5	6	5	7	7	7
7	7	7	4	1	4	7	4
6	6	6	6	5	6	7	4
5	5	4	5	5	5	4	4
7	7	7	7	1	7	7	7
1	1	1	7	1	4	7	2
7	7	7	7	7	7	7	7
1	1	5	7	2	7	7	4
5	7	7	7	1	7	7	7
5	4	7	7	1	7	7	7
1	1	1	6	1	5	7	5
2	3	3	5	4	5	7	7
5	1	4	6	5	7	7	6
4	1	7	7	1	7	7	7
2	3	1	7	4	7	7	2
1	1	1	6	4	6	5	5
4	3	4	7	2	7	7	7
3	3	2	6	5	5	3	4
6	6	6	7	5	5	7	7
7	7	7	7	7	7	7	7
6	4	7	7	4	7	7	7
4	3	2	6	5	5	5	5
1	1	1	7	1	1	7	7
2	2	6	5	3	5	5	7
6	7	7	7	4	5	5	1
4	4	7	5	4	4	7	5
2	1	2	7	4	5	7	6
5	5	5	5	4	4	5	5
4	4	3	5	3	4	4	3
4	4	3	5	2	4	4	3
3	4	4	4	3	3	3	3
3	2	2	5	4	3	4	4
5	5	6	6	5	6	4	6
4	3	4	4	3	3	4	5
5	5	1	5	5	5	2	4
5	5	1	4	5	5	5	4
4	4	4	5	4	4	4	4
7	7	3	7	5	4	4	4
6	1	3	1	1	1	6	3
7	1	3	6	5	5	6	3
4	5	3	5	4	5	5	5
7	1	3	6	5	5	6	3
6	6	5	6	6	6	7	4

Data generated or analyzed during this study

5	7	7	7	1	7	7	5
5	5	6	6	6	6	4	1
1	1	2	2	1	1	1	7
1	1	2	2	1	1	1	1
5	5	6	6	6	6	4	1
5	5	6	6	6	6	4	1
5	4	5	5	4	6	6	4
5	5	6	6	6	6	4	1
1	1	1	3	3	5	5	3
4	5	2	4	5	2	2	4
6	6	6	6	6	6	6	6
6	7	6	6	6	7	6	7
7	7	7	7	7	4	4	4
7	5	6	6	5	5	7	7
7	7	7	7	1	1	1	7
6	6	6	5	1	6	5	6
6	6	6	6	6	6	7	6
1	1	1	7	5	1	7	5
1	1	1	5	1	5	7	7
5	6	6	6	2	6	5	4
7	7	7	4	1	7	7	7
5	6	6	6	5	6	5	6
7	1	7	7	1	7	7	7
6	1	6	7	6	6	6	6
1	1	5	7	6	6	6	5
5	7	7	7	1	7	7	7
5	4	5	5	5	5	6	4
6	6	4	6	6	6	6	5
7	5	7	7	4	6	6	4
5	5	5	7	7	7	7	5
7	6	7	6	6	6	6	6
6	1	4	4	4	5	4	5
6	1	4	4	3	6	3	5
3	4	4	6	5	4	4	5
4	6	6	5	5	6	5	4
7	7	7	7	1	6	7	6
1	1	1	1	2	6	7	6
3	3	3	5	4	4	4	4
7	7	7	7	1	7	7	7
5	4	3	6	6	6	7	7
7	3	6	1	7	3	1	5
5	5	5	5	5	5	7	7
5	4	6	7	5	6	6	5
7	7	5	7	7	7	7	6
1	1	4	4	1	7	7	3
5	4	4	5	5	6	7	7
6	2	1	2	4	6	3	5
2	1	1	6	5	5	6	6
1	2	1	7	1	6	6	4
4	1	5	7	2	7	6	5
7	7	7	7	7	7	7	7
6	4	6	7	5	7	7	5
1	1	1	7	2	7	6	6

Data generated or analyzed during this study

5	4	4	5	4	4	5	4
7	3	5	6	4	4	6	6
5	3	4	5	6	3	5	3
6	6	6	6	6	6	6	3
7	7	7	7	7	7	7	7
6	1	5	6	5	6	6	3
7	5	6	7	6	7	7	5
5	5	5	5	4	4	5	5
1	1	7	1	1	1	7	7
6	6	6	6	6	7	7	6
3	5	1	7	3	7	7	6
5	7	7	7	5	7	7	5
7	1	7	7	3	6	7	4
7	4	7	7	2	7	7	7
1	1	1	7	1	7	7	7
6	6	5	7	4	6	7	6
5	2	5	3	5	4	1	7
6	3	5	5	6	7	5	5
1	1	1	6	1	7	7	7
1	1	1	6	1	7	7	7
7	7	7	7	7	7	7	7
5	4	3	6	6	6	7	7
1	1	4	4	1	7	7	3
7	5	6	7	6	7	7	5
1	1	7	1	1	1	7	7
5	4	4	5	4	4	5	4
1	2	1	7	1	6	6	4
5	5	5	5	4	4	5	5
2	6	2	7	5	7	7	3
4	1	7	5	1	7	7	7
7	5	7	7	1	7	7	1
7	7	1	5	1	7	7	7
4	4	4	4	4	4	7	5
7	7	7	7	1	7	7	7
2	4	1	7	1	7	7	7
2	4	1	7	1	7	7	7
1	1	1	5	1	6	4	1
7	7	5	7	1	7	7	7
2	6	2	7	5	7	7	4
2	1	3	7	5	7	7	6
4	4	4	4	4	4	5	7
4	4	3	5	7	1	7	6
7	6	6	5	4	5	6	4
7	6	7	4	6	7	2	7
7	5	7	7	2	5	6	5
1	4	2	4	4	6	2	5
7	1	5	7	1	7	7	4
7	1	7	7	4	7	7	1
4	4	4	7	1	7	7	7
7	5	1	7	5	7	7	5
5	3	2	5	3	4	3	5
6	6	7	7	6	7	7	7
5	3	4	3	2	4	3	3

Data generated or analyzed during this study

2	1	1	7	2	7	7	5
5	1	1	5	5	3	7	3
2	5	6	7	7	1	7	2
5	4	4	7	7	6	6	4
1	1	5	5	1	7	7	1
5	4	7	7	1	7	7	7
7	4	4	4	2	7	7	7
1	4	7	7	5	6	7	6
6	5	5	6	5	6	6	6
6	1	1	7	1	6	7	7
6	3	7	7	2	7	7	7
5	5	3	5	2	5	5	6
1	1	2	6	4	6	6	2
4	1	4	7	4	4	6	4
5	4	4	5	3	4	4	4
5	5	4	5	4	5	5	4
5	1	5	2	1	3	6	4
1	1	1	7	6	4	4	7
5	4	5	7	1	7	7	6
5	5	6	6	6	6	5	6
5	5	6	6	6	6	4	1
7	7	7	7	1	7	7	4
2	2	2	2	2	2	3	2
5	5	5	5	1	7	7	5
4	4	1	6	2	4	4	6
4	4	5	6	5	6	7	6
5	6	5	5	4	4	6	5
6	4	3	7	3	7	1	6
1	1	7	7	1	6	7	4
6	5	6	7	3	4	7	4
6	4	5	6	2	6	6	3
1	3	6	7	4	7	7	4
7	7	7	7	1	7	7	4
7	7	7	7	6	7	7	5
7	6	7	6	6	6	7	6
7	5	7	7	1	5	7	5
7	7	7	1	5	7	7	4
5	1	7	7	7	7	7	7
6	6	6	6	5	6	5	5
4	4	4	5	4	4	4	4
4	6	6	6	3	3	7	2
7	7	1	7	1	7	7	5
1	1	4	7	1	7	7	1
7	7	7	7	7	7	7	7
5	7	7	2	4	7	6	4
5	5	5	7	7	7	7	5
4	4	5	5	3	3	2	1
3	3	3	7	1	7	6	4
4	4	4	4	4	4	4	4
1	1	7	7	1	7	7	6
7	6	6	6	5	6	6	6
5	4	1	6	2	5	5	3
5	5	5	5	7	5	6	5

Data generated or analyzed during this study

7	3	5	6	4	4	6	5
6	5	1	2	1	1	7	3
1	1	7	7	1	7	7	7
5	5	5	7	7	7	7	5
5	5	5	7	7	7	7	5
1	1	1	5	2	4	6	6
7	6	6	6	5	6	7	5
7	7	7	7	4	7	7	7
2	2	3	4	6	4	3	5
2	6	2	7	5	7	7	4

Data generated or analyzed during this study

control6	control5	control4	control3	control2	control1	subjective1	subjective9
1	7	7	7	7	7	7	7
3	6	4	6	5	1	7	7
2	4	4	5	4	6	3	4
4	5	5	5	4	4	6	6
1	7	7	7	7	7	7	7
1	7	7	7	7	7	7	7
2	1	4	6	4	4	7	4
7	5	5	7	7	6	6	6
1	7	7	7	7	7	7	7
4	3	4	5	3	5	2	4
4	5	4	2	7	7	7	7
6	4	3	3	3	1	3	4
3	5	3	5	6	6	6	7
4	4	4	4	4	4	5	5
5	3	3	5	2	5	5	7
3	7	4	7	5	5	6	7
4	3	5	5	5	4	5	4
3	5	4	4	4	4	4	3
3	4	4	5	4	3	4	4
4	4	2	4	4	5	1	4
2	1	5	5	7	7	4	5
7	1	2	4	5	7	7	7
7	6	6	7	6	7	4	7
7	7	5	5	7	7	7	7
7	3	5	1	4	5	3	4
4	4	4	5	5	4	4	4
7	6	6	4	7	7	5	7
2	4	6	5	5	4	5	4
3	5	5	5	5	5	5	4
1	7	7	7	7	7	7	7
3	4	4	4	2	3	3	5
5	4	4	5	5	5	7	6
7	7	7	6	7	1	7	6
5	5	3	5	4	4	4	5
3	3	2	6	4	5	4	4
3	6	3	2	6	4	4	7
1	7	7	3	1	7	7	7
5	4	4	6	4	4	4	5
3	4	5	5	5	6	6	4
3	4	5	6	5	6	7	4
6	5	5	3	3	6	6	6
5	4	4	4	6	4	4	3
6	5	7	3	6	6	6	6
3	3	3	5	6	4	4	5
6	2	5	5	6	6	6	2
7	2	7	1	1	2	1	3
5	6	5	3	4	5	5	7
2	6	7	6	5	3	6	5
4	4	4	4	4	4	6	6
2	3	4	3	5	4	4	4
4	3	4	5	3	3	2	3

Data generated or analyzed during this study

3	5	5	4	5	5	7	6
3	6	4	5	6	6	6	7
3	5	4	3	5	5	7	7
3	4	5	4	5	5	7	7
2	6	7	7	6	6	6	5
4	6	5	1	6	4	4	4
3	4	5	4	5	5	7	7
3	5	5	1	5	5	7	6
5	4	4	3	5	4	4	4
5	5	4	1	5	5	6	6
7	7	7	7	1	7	4	7
5	6	5	2	6	6	6	5
5	4	4	3	5	4	4	4
1	7	7	7	7	7	7	7
7	1	2	7	4	4	4	3
5	5	7	7	3	4	6	6
1	7	6	7	7	7	7	7
7	5	7	7	7	7	7	7
5	5	2	5	5	3	3	5
7	1	3	7	4	4	4	3
4	3	5	6	7	6	7	6
7	2	1	2	1	1	1	1
4	4	5	4	7	4	4	1
1	4	5	7	6	7	6	7
7	4	6	7	6	6	7	6
7	6	7	7	7	7	7	7
3	5	7	7	7	7	6	2
5	4	3	4	4	5	2	4
4	5	5	5	5	5	5	6
2	6	6	6	6	6	6	6
1	6	5	6	5	6	6	5
7	6	6	5	6	6	6	5
4	4	4	4	4	4	4	4
1	7	7	7	7	7	7	7
3	4	5	3	3	4	2	1
5	2	3	3	3	1	5	2
4	5	6	5	6	5	5	3
4	4	4	4	4	4	4	4
2	6	7	6	5	6	5	6
3	6	6	5	7	7	4	7
2	6	7	6	5	6	5	6
2	6	7	6	5	6	5	6
1	7	7	7	7	7	7	7
6	4	5	4	5	5	7	4
6	6	6	7	6	7	6	6
4	7	3	4	2	7	7	7
4	4	4	4	4	4	4	7
6	4	2	6	7	6	4	7
2	4	6	6	5	6	4	5
2	6	6	6	7	7	2	7
4	6	4	5	3	5	4	2
5	4	2	4	4	3	4	4
7	2	3	1	7	1	3	2

Data generated or analyzed during this study

6	6	6	7	6	7	6	6
1	7	7	7	7	7	7	7
7	7	7	7	7	7	7	7
1	7	7	7	7	7	7	7
5	5	4	4	5	5	5	6
1	7	7	7	6	5	3	5
3	5	4	5	6	7	4	6
7	5	7	7	1	4	1	1
7	5	7	7	1	4	1	1
4	5	5	3	6	5	6	6
7	5	5	5	6	7	7	7
7	6	5	7	1	7	7	7
6	5	6	6	6	6	4	7
4	4	3	5	4	5	5	4
7	7	6	5	7	7	4	7
7	5	4	1	7	7	1	4
7	7	7	7	6	7	6	7
4	5	4	2	7	7	7	7
7	7	7	6	1	7	7	7
7	7	7	7	7	7	7	7
5	5	4	4	4	5	1	6
3	5	5	2	4	5	5	4
7	6	6	5	2	4	7	6
7	5	7	5	5	7	1	7
1	5	4	3	7	7	7	6
1	6	5	3	7	7	7	6
1	6	5	1	1	7	7	7
5	5	4	5	4	5	6	2
5	6	6	6	6	7	5	6
1	7	7	7	7	7	7	7
7	7	7	7	7	7	7	7
4	3	5	5	5	4	5	4
4	4	2	4	4	5	1	4
7	6	7	7	1	3	5	6
7	1	2	4	5	7	7	7
7	6	5	5	5	6	4	5
7	4	6	6	2	4	6	4
3	5	5	5	5	5	5	5
4	4	4	3	5	5	4	3
4	4	4	3	5	5	4	3
4	4	4	4	5	5	3	3
3	4	4	4	5	5	3	3
7	5	7	4	4	6	7	7
3	5	6	6	6	2	3	4
3	2	3	3	4	3	6	5
7	3	4	5	4	4	5	4
3	5	5	5	4	4	4	3
7	5	5	6	5	5	5	5
7	3	7	2	1	4	1	1
6	5	4	5	7	3	5	5
3	5	4	4	5	4	5	5
6	5	4	5	7	3	5	5
7	7	3	6	6	6	6	6

Data generated or analyzed during this study

4	6	7	5	7	7	7	7
1	7	7	7	6	7	6	6
4	7	5	1	7	7	1	7
4	7	5	1	7	7	1	7
1	7	7	7	6	7	6	6
4	4	4	4	6	4	6	6
7	6	6	4	5	6	6	5
1	7	7	7	6	7	6	6
3	2	5	5	7	2	6	4
6	3	4	5	3	1	2	3
2	6	6	6	6	6	6	6
1	5	7	6	7	6	7	5
1	4	7	7	7	7	7	7
1	7	7	4	7	7	7	7
2	6	5	3	1	5	6	7
2	6	6	6	7	6	5	7
2	6	6	6	6	6	6	6
1	4	5	6	5	4	7	4
7	6	7	7	7	4	7	7
3	4	5	6	5	6	5	3
7	7	7	7	4	7	1	7
2	7	5	4	5	5	5	3
7	7	7	7	7	7	7	7
7	4	7	4	5	7	5	5
6	4	3	5	4	4	4	7
7	7	7	7	7	7	5	3
7	3	4	3	4	6	6	4
5	5	5	3	6	4	4	5
6	6	4	7	7	7	3	6
1	5	4	4	4	5	7	7
1	6	5	6	6	6	6	7
2	4	5	7	4	5	7	4
2	4	5	7	3	6	7	4
3	5	4	4	3	3	3	3
4	6	5	5	4	5	6	5
2	5	4	5	6	5	7	7
5	5	5	6	5	6	6	6
4	4	5	4	5	4	3	3
7	6	4	1	7	7	7	7
2	6	6	5	6	5	5	5
2	2	3	6	1	5	3	1
3	5	5	5	5	5	5	5
2	5	6	6	7	6	6	6
2	4	4	2	1	2	7	6
7	4	5	7	7	4	1	7
6	6	6	7	6	7	6	6
3	5	5	5	5	5	7	6
7	2	6	5	6	4	4	4
7	3	2	1	7	1	5	3
7	4	4	3	5	7	4	3
1	7	7	7	7	7	7	7
3	7	2	6	3	5	4	7
2	6	6	5	7	6	5	1

Data generated or analyzed during this study

4	5	5	5	3	5	4	4
6	6	7	6	6	5	7	7
5	5	2	5	6	3	5	3
2	2	6	6	6	6	6	6
7	7	7	7	1	7	7	7
2	5	4	2	3	6	1	5
4	4	6	5	4	5	7	7
4	3	4	4	4	4	5	5
7	1	7	7	1	1	7	1
2	6	6	6	6	6	6	6
4	6	6	6	6	6	6	6
5	7	7	7	6	7	5	7
5	7	7	7	1	7	7	7
3	6	5	5	7	7	7	7
1	6	3	2	5	7	7	7
3	6	6	5	7	7	7	6
1	7	3	1	3	4	6	2
3	7	4	4	6	6	5	5
7	5	6	4	1	5	5	7
7	5	6	4	1	5	5	7
1	7	7	7	7	7	7	7
2	6	6	5	6	5	5	5
7	4	5	7	7	4	1	7
4	4	6	5	4	5	7	7
7	1	7	7	1	1	7	1
4	5	5	5	3	5	4	4
7	3	2	1	7	1	5	3
4	3	4	4	4	4	5	5
1	5	5	7	7	5	7	7
1	7	7	7	7	7	7	2
7	7	7	7	7	7	7	7
4	4	4	4	4	4	7	7
1	7	4	2	6	7	4	4
7	7	7	7	7	7	7	7
7	7	7	7	7	7	7	7
7	7	7	7	7	7	7	7
1	5	1	7	7	7	1	7
7	7	7	7	5	7	7	7
1	5	5	7	7	5	7	7
6	6	5	5	5	7	7	6
4	4	4	4	4	4	4	4
4	4	4	4	4	5	3	3
2	3	7	5	3	4	5	3
1	7	7	5	6	7	7	5
3	7	7	5	7	5	7	5
6	6	4	5	5	5	7	7
7	7	7	7	7	7	7	7
7	7	7	7	7	7	7	7
7	7	5	7	4	7	7	7
7	4	4	3	7	5	7	7
4	4	4	5	4	4	3	4
6	6	5	5	4	7	7	7
4	4	6	4	5	5	5	4

Data generated or analyzed during this study

7	2	3	1	7	1	3	2
6	4	5	5	6	3	3	7
1	5	6	5	7	7	7	7
4	4	6	3	2	4	5	4
7	7	6	6	5	7	7	7
7	7	7	7	7	7	7	7
1	4	3	7	1	4	7	1
7	7	6	6	6	7	7	7
2	5	5	5	6	6	7	5
6	6	6	6	1	7	7	7
7	6	5	6	5	7	2	6
7	4	3	5	5	3	3	3
1	6	5	5	7	7	7	6
4	4	1	4	7	4	4	4
4	4	4	4	4	5	3	4
4	5	4	5	5	4	4	5
6	3	4	4	4	4	3	3
1	1	5	4	7	4	7	7
6	5	5	5	1	6	2	5
1	7	7	7	6	7	6	6
1	7	7	7	6	7	6	6
4	5	7	7	7	7	3	7
5	3	2	2	2	2	2	2
7	5	5	1	7	5	5	7
4	6	4	2	2	5	4	7
4	4	4	4	4	4	4	7
1	5	6	7	5	7	6	6
4	4	5	6	6	5	7	5
7	6	6	7	5	6	7	7
3	5	5	5	5	6	2	6
6	5	6	6	7	7	3	6
7	7	7	7	4	6	7	7
7	7	7	2	7	7	7	7
1	6	5	6	6	5	6	7
2	6	7	7	7	6	6	6
3	7	7	5	7	5	7	5
7	7	7	7	7	7	7	7
7	7	5	7	7	7	7	7
1	5	6	6	6	7	5	6
5	4	4	4	4	4	5	5
7	5	7	7	6	6	6	7
7	7	1	7	7	5	7	7
7	7	5	7	7	7	7	7
1	6	7	7	7	7	6	6
3	7	6	5	6	6	6	5
1	5	4	4	4	5	7	7
6	3	2	2	2	2	1	2
7	1	7	7	3	3	7	1
4	4	4	4	4	4	4	4
4	4	7	7	4	7	4	4
7	7	6	7	7	6	6	6
3	2	5	4	6	7	7	6
2	5	5	5	6	6	4	5

Data generated or analyzed during this study

4	4	4	4	4	4	7	7
7	5	5	7	7	2	1	1
7	7	7	7	2	7	7	7
1	5	4	4	4	5	7	7
1	5	4	4	4	5	7	7
6	3	4	4	7	6	6	1
6	6	6	6	7	6	7	7
1	7	7	7	7	7	7	7
7	5	2	7	7	7	7	1
1	5	5	7	7	5	7	7

Data generated or analyzed during this study

attitude	intention4	intention3	intention2	intention1	control9	control8	control7
6.57	7	7	7	7	7	7	1
6.43	4	3	3	2	5	5	3
4.5	4	5	4	4	5	4	3
5.07	7	4	6	4	4	5	2
6.57	1	7	7	7	7	7	1
6.64	1	7	7	7	2	7	1
5.07	7	6	4	1	4	5	4
6.07	3	7	7	7	4	2	1
6.57	1	7	7	7	7	7	1
5	6	5	4	4	4	3	6
6	6	7	7	7	1	7	4
2.93	2	2	3	1	1	1	5
6.14	6	7	6	6	5	6	2
5.64	4	4	4	4	5	3	3
5	7	5	4	6	5	3	3
5.57	1	2	7	6	4	4	2
5.21	4	4	4	2	3	2	4
4	4	4	4	4	5	3	3
4.36	5	2	4	4	3	5	3
6.21	1	7	7	4	4	4	4
4.64	4	5	6	5	5	5	2
5.57	2	5	3	2	4	5	7
6.86	7	7	7	7	3	3	4
6.43	7	7	7	7	6	7	1
4.79	6	3	3	3	4	4	2
3.86	4	4	4	4	4	4	4
5.57	5	7	7	7	3	5	2
5	5	4	6	4	5	5	4
5.71	3	5	5	5	5	5	3
5.93	7	7	7	7	7	7	1
5.07	5	2	2	2	4	3	5
6.57	4	5	2	3	5	7	4
5.57	2	7	7	7	7	6	2
4.14	5	4	3	6	3	5	3
4.5	5	4	6	4	5	5	3
5.64	4	4	5	5	4	6	2
7	7	7	7	6	7	4	1
5.64	4	5	5	4	4	3	4
4.86	6	5	3	3	5	3	5
5.29	3	6	3	5	5	3	3
6.29	7	6	6	7	2	1	7
4.36	4	4	4	3	5	3	7
6	3	5	6	6	6	5	2
5.07	4	5	4	3	3	5	3
4.21	6	4	4	4	4	2	6
6.64	2	5	3	5	3	1	1
5.93	6	7	6	6	5	7	3
5.07	2	6	4	4	5	6	2
4.64	3	5	3	5	4	4	4
4	5	5	5	5	5	5	3
3.93	3	5	3	3	3	3	4

Data generated or analyzed during this study

5.5	4	6	5	5	5	6	3
5.71	5	6	6	6	4	5	5
7	6	5	5	5	4	4	7
5.14	6	5	4	5	4	4	7
5.14	2	5	5	6	6	6	2
3.29	2	1	2	3	1	5	3
5.14	6	5	4	5	4	4	7
5.5	4	6	5	5	5	6	3
4.14	5	4	4	5	4	4	3
5.86	5	5	5	5	7	3	4
4.56	1	7	7	7	7	7	1
5.67	6	5	6	6	5	3	3
4.14	5	4	4	5	4	4	3
6.36	1	7	7	7	7	7	1
5.64	3	5	6	3	4	4	7
5.93	7	6	6	6	3	4	3
7	7	7	6	6	6	5	2
5.71	7	7	1	1	1	7	7
3.64	6	5	3	5	4	3	3
5.64	3	5	6	3	4	4	7
6.21	4	6	4	4	4	4	3
2.36	6	1	1	2	2	1	7
5.14	4	6	4	4	4	4	3
6.14	5	4	5	6	5	6	2
5.21	7	5	5	7	6	4	7
6.57	1	7	7	7	7	4	2
5.79	7	5	6	5	7	2	3
5	5	4	3	4	5	4	5
5.79	4	7	6	5	6	5	3
5.57	2	6	6	5	6	6	2
4.64	3	5	4	6	5	5	3
4.93	7	6	6	6	3	3	6
3.71	3	4	4	4	4	4	4
6.57	7	7	7	7	7	7	1
4.57	7	3	2	1	4	4	2
2.93	2	2	3	3	3	2	6
4.5	4	5	5	6	5	5	3
4.07	4	4	5	4	4	4	4
5.36	3	5	5	6	6	6	1
4.43	3	5	6	5	6	5	3
5.36	3	5	5	6	6	6	1
5.36	3	5	5	6	6	6	1
6.14	1	7	7	7	7	7	1
4.57	4	5	4	5	4	5	6
5.43	7	7	7	6	5	1	7
5.14	3	3	5	3	4	4	5
5.79	4	4	4	4	4	4	4
5.86	4	4	4	4	4	3	6
5.57	4	4	4	4	4	6	2
5.71	6	6	6	6	2	2	1
5.86	3	5	3	4	4	4	3
3.36	5	4	3	4	3	4	5
1.36	7	1	1	1	1	7	4

Data generated or analyzed during this study

6.93	7	7	7	6	5	1	7
6.14	1	7	7	7	7	7	1
6.57	7	7	7	7	1	1	1
4.86	1	7	7	7	7	7	1
4.21	5	5	5	5	4	3	3
4.79	7	6	5	6	4	7	2
6.86	6	5	5	6	6	6	1
7	7	1	7	7	4	4	7
7	7	1	7	7	4	4	7
5.79	4	6	4	5	5	6	3
5.79	3	6	6	5	5	6	1
6.5	7	7	7	7	3	1	7
6	2	6	6	6	4	5	7
5.64	6	5	5	4	4	4	5
6.57	7	7	7	7	1	1	1
6.36	4	1	3	3	1	1	1
6.93	7	7	7	7	7	4	6
6	6	7	7	7	1	7	4
7	7	7	7	7	7	4	7
6.57	7	7	7	7	7	1	1
5.29	5	6	5	5	4	3	4
5.43	4	5	7	7	4	6	7
6.07	7	6	3	6	5	5	6
6.71	7	7	7	7	5	1	7
6.36	7	5	4	4	1	7	1
6.36	4	5	3	3	1	7	1
5.64	7	7	3	5	7	5	2
3.5	4	3	4	5	4	4	3
6.14	5	5	4	4	7	6	5
6.57	1	7	7	7	7	7	1
6.93	7	7	7	7	7	7	6
4.86	4	4	4	2	3	5	4
5.57	1	7	7	4	4	4	4
5.64	7	4	5	4	5	6	7
5.57	2	5	3	2	4	5	7
5.79	7	6	6	5	2	2	5
6.14	2	6	6	6	1	1	2
4.29	4	4	4	4	5	5	3
4	4	4	4	3	3	4	4
4	4	4	4	3	3	4	4
3.64	4	4	3	3	3	4	4
4.21	4	4	3	3	4	5	3
6.43	4	4	5	5	5	6	3
3.29	3	3	5	4	4	5	2
5.29	7	4	3	4	5	4	3
5.93	4	4	4	5	5	2	4
4.43	5	5	4	4	5	4	4
6.36	5	2	3	5	5	3	5
4.71	6	2	2	3	5	1	7
5.07	6	6	4	4	6	5	6
4.29	4	4	4	4	5	5	3
5.07	2	6	4	4	6	5	6
6.07	6	6	6	6	1	6	6

Data generated or analyzed during this study

6.71	7	7	4	5	1	7	2
5.64	2	6	5	5	7	7	1
6.57	7	7	7	7	4	1	1
6.57	7	7	7	7	4	1	1
5.64	2	6	5	5	7	7	1
5.64	1	1	7	7	7	4	4
5.36	4	6	6	5	4	2	2
5.64	2	6	5	5	7	7	1
5.43	2	2	5	5	6	5	5
3.86	2	2	3	3	4	3	4
5.43	2	6	6	6	6	6	2
5.86	1	6	7	6	7	7	2
6.21	4	5	5	5	1	7	7
6.14	1	7	7	7	7	5	2
6.57	2	6	5	5	5	5	3
6.14	6	6	6	6	5	5	2
5.36	7	6	6	5	5	6	1
4.64	3	6	3	4	1	5	4
5.64	7	5	4	4	7	7	1
6.14	7	5	4	6	5	5	2
6.93	7	7	7	7	4	4	1
5.93	5	6	6	6	5	3	4
7	7	7	7	7	7	1	7
5.14	2	6	7	7	6	6	3
5.21	7	6	4	2	4	2	7
7	7	7	7	7	1	1	1
5.36	3	5	4	4	5	3	3
5.36	3	5	6	6	6	5	4
6.5	7	7	6	7	6	7	5
6.79	6	7	5	5	2	5	4
5.43	6	7	6	6	5	5	6
4.86	5	7	4	5	3	3	5
4.14	5	7	4	5	3	3	5
3.93	4	4	4	4	4	3	4
6.43	4	5	6	6	5	5	2
6.57	4	5	6	5	5	6	3
6.64	5	5	5	5	1	4	5
4.07	4	4	4	4	4	4	3
4.86	7	7	7	7	2	7	3
4.86	2	6	5	5	6	5	2
3.93	7	5	3	5	2	6	7
6.29	3	5	5	5	5	5	3
6.43	7	6	5	6	3	4	2
3.79	3	6	1	1	5	6	7
6.07	7	1	7	4	1	1	1
6.86	7	7	7	6	5	1	7
5.43	4	6	5	5	5	6	2
5.86	3	3	1	3	3	2	5
1.64	7	3	2	2	1	7	2
2.86	6	7	5	5	6	2	5
5.07	1	7	7	7	7	7	1
5.79	6	7	7	5	6	2	3
6.14	6	6	6	6	6	2	2

Data generated or analyzed during this study

4	3	4	4	5	4	4	3
5	6	7	5	5	5	5	1
4.36	4	4	4	4	4	5	3
5.64	2	6	6	6	6	7	2
6.93	7	7	7	7	7	1	7
6.57	3	5	4	4	2	1	4
5.86	7	6	5	4	4	6	6
4.57	3	3	3	4	4	4	4
6.57	7	1	7	7	1	1	7
5.64	1	7	7	7	7	6	2
6.21	6	6	6	6	4	2	4
5.5	7	6	7	7	6	5	5
6.36	4	4	7	7	3	4	7
6.57	6	7	7	7	4	4	7
5.64	7	7	7	7	3	4	7
4.43	3	5	6	5	6	5	3
6.07	5	4	6	7	4	7	1
5.14	3	5	6	5	6	4	4
4.5	7	5	5	3	2	2	4
4.5	7	5	5	3	2	2	4
5.71	1	7	7	7	7	7	1
4.86	2	6	5	5	6	5	2
6.07	7	1	7	4	1	1	1
5.86	7	6	5	4	4	6	6
6.57	7	1	7	7	1	1	7
4	5	4	4	5	4	4	3
3.45	7	3	2	2	1	7	2
4.57	3	3	3	4	4	4	4
6.21	7	7	5	5	2	4	1
6	7	7	7	7	7	1	1
6.21	7	7	7	7	5	6	7
5.93	7	7	7	7	7	4	4
4.86	7	6	7	7	4	2	1
7	3	5	7	4	7	1	7
6	7	7	7	7	7	1	1
6.43	7	7	7	7	7	1	1
6.43	7	3	7	7	1	1	4
7	7	6	7	7	4	1	4
6.21	7	7	5	5	2	4	1
5.29	6	6	6	7	2	7	4
4.64	7	5	5	5	7	4	4
4.79	5	4	4	3	5	4	3
6.21	7	4	3	3	5	3	3
5.57	2	5	6	7	6	7	2
5.86	3	5	5	5	5	5	3
5.21	7	7	7	5	5	4	6
6.79	7	1	7	7	4	4	7
7	7	7	7	7	7	7	4
6.36	7	7	7	7	7	7	7
6.29	4	7	4	4	2	6	1
4.21	4	5	4	4	4	3	4
6.5	2	5	6	6	3	4	2
5.29	6	6	5	5	5	4	4

Data generated or analyzed during this study

1.43	7	1	1	1	1	7	4
3.86	6	2	2	1	5	5	3
3.32	6	6	6	5	5	5	1
5.65	3	5	5	3	4	3	7
7	7	7	7	7	1	3	3
6.57	7	7	7	7	7	1	1
7	4	4	4	4	1	1	1
6.57	7	7	7	7	1	3	4
6.07	3	5	5	5	6	5	2
6.71	7	6	6	7	1	1	3
5.79	6	7	7	7	2	1	7
5.21	6	5	5	5	1	2	7
6.36	4	5	3	3	1	7	2
4.71	4	4	4	4	4	4	4
4.43	4	4	5	4	4	4	3
4.43	4	5	4	4	5	5	3
4.43	5	4	4	4	4	3	2
7	5	7	7	7	7	5	1
6.71	4	5	6	6	5	5	3
5.36	2	6	5	5	7	7	1
5.64	2	6	5	5	7	7	1
6.57	7	7	7	7	7	1	1
2.64	6	2	3	3	2	2	6
6.64	3	7	5	5	1	1	3
5.36	2	7	7	7	7	3	4
6.64	7	7	7	7	7	4	4
6.71	7	6	7	6	6	2	6
4.57	2	5	5	5	5	6	4
5.5	7	7	5	5	1	1	7
5.29	3	7	5	5	5	5	4
4.27	6	6	6	6	5	2	4
5.93	7	7	7	7	6	6	2
6.79	7	7	7	7	5	1	7
5.93	3	7	7	5	6	4	1
5.86	2	7	6	7	6	7	2
5.86	3	5	5	5	5	5	3
6.14	1	7	7	7	7	5	1
6.43	7	5	5	7	5	7	1
6	5	6	6	7	5	6	2
5.36	3	4	4	4	4	3	4
6.07	6	6	5	6	3	2	2
6.5	7	7	7	7	7	7	1
6	7	7	7	7	4	1	7
6.57	2	7	7	7	7	7	1
5.71	6	7	7	7	7	6	2
6.79	6	7	5	5	2	5	6
3.79	5	3	4	5	4	2	8
2.34	1	7	2	1	1	7	7
3.93	4	4	5	4	4	4	4
6.86	7	7	7	7	4	4	4
6.57	3	6	7	7	7	5	6
6.79	6	3	3	4	6	1	1
4.07	2	6	6	6	5	4	3

Data generated or analyzed during this study

4.64	6	7	5	5	5	4	4
7	6	6	5	5	5	1	7
6.86	7	7	7	7	7	5	1
6.79	6	7	5	5	2	5	4
6.79	6	7	5	5	2	5	4
5.14	7	7	1	1	2	4	5
6.36	7	7	6	6	5	4	7
6.57	1	7	7	7	7	7	1
5.29	7	6	7	7	2	1	7
6.57	7	7	5	5	2	4	1

Data generated or analyzed during this study

behavior12	behavior1r	patientcove	workeexpe	intention	perceivedb	subjectiver
2	2	2	1	7	5.67	7
1	1	1	1	3	4.22	4.5
1	1	2	1	4.25	4.11	4
1	1	1	1	5.25	4.22	4.8
1	1	1	1	5.5	5.67	7
2	2	1	1	5.5	5.11	6.34
1	1	1	1	4.5	3.78	3.9
2	2	1	1	6	4.89	4.9
2	2	1	1	5.5	5.67	7
1	1	1	1	4.75	4.11	2.7
2	2	1	1	6.75	4.56	5.3
1	1	1	1	2	3	2.8
1	1	1	1	6.25	4.56	5.7
1	1	1	1	4	3.89	4.9
1	1	1	1	5.5	3.78	5.1
1	1	1	1	4	4.56	5.8
1	1	1	1	3.5	3.89	4.4
1	1	1	1	4	3.98	4.1
1	1	2	1	3.75	3.78	4.1
1	1	1	1	4.75	3.89	2.5
2	2	1	1	5	4.33	6.1
2	2	3	1	3	4.67	5.6
1	1	3	1	7	5.44	3.6
2	2	2	1	7	5.78	6.5
2	2	2	1	3.75	3.89	4
1	1	1	1	4	4.22	3.7
1	1	1	1	6.5	5.22	4.8
1	1	3	1	4.75	4.44	4.3
1	1	3	1	4.5	4.56	4.7
1	1	1	1	7	5.67	7
2	2	2	1	2.75	3.56	4.76
1	1	1	1	3.5	4.89	5.1
1	1	1	1	5.75	5.56	5.3
2	2	1	1	6.1	4.11	4.2
2	2	1	1	4.75	4	3.9
1	1	1	1	4.5	4	4.5
2	2	2	1	6.75	4.22	7
1	1	1	1	4.5	4.22	3.9
2	2	2	1	4.25	4.56	5.2
1	1	2	1	4.25	4.44	5
1	1	1	1	6.5	4.22	5.8
1	1	1	1	3.75	4.67	4.9
1	1	1	1	5	5.11	5.8
1	1	2	2	4	3.89	4.4
1	1	1	1	4.5	4.67	3.8
2	2	1	1	3.75	2.78	3.6
1	1	1	1	6.25	4.78	6
2	2	1	1	4	4.67	5.6
1	1	1	1	4	4	5.7
2	2	1	1	5	3.78	3.7
1	1	1	1	3.5	3.56	3.6

Data generated or analyzed during this study

2	2	1	1	5	4.56	3.9
2	2	1	1	5.75	4.89	5.3
1	1	2	1	5.25	4.44	5.3
1	1	2	1	5	4.56	5.1
1	1	1	1	4.5	5.33	5.8
2	2	2	1	2	3.89	4.2
1	1	2	1	5	4.56	5.1
2	2	1	1	5	5.11	3.9
2	2	1	1	4.5	4	4.3
2	2	1	1	5	4.33	4.2
1	1	1	1	5.5	5.67	6
2	2	1	1	5.75	4.56	5.1
2	2	1	1	4.5	4	4.3
1	1	1	1	5.5	5.67	5.8
2	2	1	1	4.25	4.44	4.6
1	1	1	1	6.25	4.56	5.5
2	2	1	1	6.5	5.33	6.1
1	1	1	1	4	6.11	5.2
1	1	1	1	4.75	3.89	4
2	2	1	1	4.25	4.56	4.6
1	1	1	1	4.5	4.67	4.3
1	1	1	1	2.5	2.67	1
1	1	1	1	4.5	4.33	5
1	1	1	1	5	4.78	4
1	1	1	1	6	5.89	5.5
1	1	1	1	5.5	6	7
2	2	1	1	5.75	5.33	5.1
1	1	1	1	4	4.33	3.8
1	1	1	1	5.5	4.78	4.6
1	1	1	1	4.75	5.11	6
1	1	1	1	4.5	4.67	5.3
2	2	3	2	6.25	5.33	4
1	1	2	1	3.75	4	3.9
2	2	2	1	7	5.67	7
1	1	2	2	3.25	3.56	3.9
1	1	1	2	2.5	3.11	3
1	1	2	1	5	4.89	4.6
1	1	1	2	4.25	4	4
1	1	1	2	4.75	5	5.6
2	2	1	2	4.75	5.33	5.8
1	1	1	2	4.75	5	5.6
1	1	1	2	4.75	5	5.6
1	1	1	1	5.5	5.67	6
1	1	1	1	4.5	4.89	4.2
1	1	1	1	6.75	5.67	5.6
2	2	1	2	3.5	4.44	4.7
1	1	1	2	4	4	5
1	1	1	1	4	4.89	6.4
1	1	1	2	4	4.56	5.9
1	1	1	1	6	4.33	6
2	2	1	2	3.75	4.22	3.7
2	2	1	2	4	3.78	3.6
1	1	1	2	2.5	3.67	3.1

Data generated or analyzed during this study

1	1	1	1	6.75	5.67	5.6
2	2	1	1	5.5	5.67	7
1	1	1	1	7	5	5
2	2	1	1	5.5	5.67	7
2	2	1	2	5	4.22	5.3
2	2	1	2	6	5.11	5.5
1	1	1	1	5.5	4.78	6.4
1	1	1	2	5.5	5.11	2.7
1	1	1	2	5.5	5.11	2.7
1	1	1	2	4.75	4.67	5.6
1	1	1	2	5	5.22	6.2
2	2	1	1	7	4.89	5.5
2	2	1	2	5	5.67	5.7
2	2	1	2	5	4.22	4.6
2	2	1	2	7	4.67	6.1
2	2	1	2	2.75	3.78	2.9
1	1	1	1	7	6.44	6.9
2	2	1	2	6.75	4.56	4.8
1	1	1	2	7	5.89	6.2
1	1	2	2	7	5.67	5.9
1	1	1	1	5.25	4.22	3.4
1	1	1	1	5.75	4.56	4.5
1	1	1	1	5.5	5.11	5.4
1	1	1	2	7	5.44	4.9
2	2	1	2	5	4	4.6
2	2	1	2	3.75	4.22	4.2
2	2	1	2	5.5	3.89	5.5
2	2	1	1	4	4.33	3.9
2	2	1	1	4.5	6	6
2	2	1	1	5.5	5.67	7
1	1	1	2	7	6.89	6.3
1	1	1	2	3.5	4.22	4.4
1	1	1	2	4.75	3.89	3.1
1	1	1	2	5	5.44	4.6
2	2	3	1	3	4.67	5.6
2	2	1	2	6	4.78	4.9
1	1	1	1	5	3.67	4.4
2	2	1	2	4	4.56	4.8
1	1	1	2	3.75	4	3.7
1	1	1	2	3.75	4	3.6
1	1	1	1	3.5	4.11	3.3
1	1	1	1	3.5	4.11	3.3
1	1	2	2	4.5	5.22	5.7
2	2	1	1	3.75	4.33	3.7
1	1	2	2	4.5	3.33	4.3
2	2	1	1	4.25	4.22	4.3
1	1	2	1	4.5	4.33	4
1	1	1	2	3.75	5.11	5.1
1	1	1	1	3.25	4.11	2.4
1	1	2	2	5	5.22	4.6
1	1	1	2	4	4.22	4.6
1	1	2	2	4	5.22	4.6
1	1	2	2	6	5.33	5.8

Data generated or analyzed during this study

1	1	1	2	5.75	5.11	6
1	1	1	1	4.5	5.56	5.1
1	1	1	2	7	4.11	2.4
2	2	1	1	7	4.11	1.8
1	1	1	1	4.5	5.56	5.1
1	1	1	2	4	4.56	5.1
1	1	1	2	5.25	4.67	5
1	1	1	1	4.5	5.56	5.1
1	1	1	2	3.5	4.44	3.2
2	2	1	2	2.5	3.67	3.3
1	1	1	1	5	5.11	6
1	1	1	1	5	5.33	6.3
2	2	1	1	4.75	5.33	6.1
2	2	2	1	5.5	5.22	6.2
1	1	1	2	4.5	3.89	5.1
1	1	2	2	6	5	5.3
2	2	2	2	6	4.89	6.1
2	2	2	2	4	3.89	3.9
2	2	1	2	5	5.89	4.2
1	1	1	2	5.5	4.56	4.8
2	2	2	2	7	5.33	5.5
2	2	1	2	5.75	4.44	5.3
2	2	2	2	7	6.33	5.8
1	1	2	2	5.5	5.44	5.4
2	2	1	2	4.75	4.33	4.8
2	2	1	2	7	4.56	5.6
2	2	1	1	4	4.22	4.9
1	1	1	2	5	4.78	5.4
1	1	1	1	6.75	6.11	5.5
2	2	1	2	5.75	3.78	6.2
2	2	1	2	6.25	5.11	6.3
2	2	1	1	5.25	4.22	4.4
2	2	1	2	5.25	4.22	4.3
1	1	1	1	4	3.67	4.1
1	1	1	2	5.25	4.56	5.2
2	2	2	1	5	4.56	6.2
2	2	1	2	5	4.67	3.7
1	1	1	2	4	4.11	3.6
1	1	1	2	7	4.89	6.4
2	2	1	2	4.5	4.78	5.4
1	1	1	2	5	3.78	3.7
2	2	1	1	4.5	4.56	5.4
1	1	1	2	6	4.56	5.6
2	2	1	2	2.75	3.67	6.6
1	1	1	2	4.75	4.11	3.6
1	1	1	2	6.75	5.67	5.5
1	1	1	2	5	4.56	4.2
2	2	1	1	2.5	4.44	4
1	1	1	2	3.5	3.44	3.6
1	1	1	1	5.75	4.78	4.4
1	1	1	1	5.5	5.67	7
1	1	1	2	6.25	4.11	5.8
2	2	1	2	6	4.67	3.7

Data generated or analyzed during this study

1	1	1	2	4	4.22	4.3
1	1	2	1	5.75	5.22	5.5
1	1	1	2	4	4.22	4.2
1	1	1	2	5	4.78	5.7
2	2	1	2	7	5.67	7
1	1	1	1	4	3.22	4.4
2	2	2	2	5.5	4.89	6.4
1	1	2	1	3.25	3.89	4.8
1	1	1	2	5.5	3.67	3.4
2	2	2	2	5.5	5.22	6.2
2	2	2	2	6	4.89	5.1
2	2	1	2	6.75	6.11	6.2
1	1	1	2	5.5	5.33	5.6
1	1	1	2	6.75	5.33	6.2
2	2	2	1	7	4.22	4.6
2	2	1	2	4.75	5.33	6
2	2	1	2	5.5	3.44	4
1	1	1	2	4.75	4.89	5.2
2	2	2	2	5	4	4.3
2	2	2	2	5	4	4.3
1	1	1	2	5.5	5.67	7
2	2	1	2	4.5	4.78	5.4
1	1	1	2	4.75	4.11	3.6
2	2	2	2	5.5	4.89	6.4
1	1	1	2	5.5	3.67	3.4
1	1	1	2	4.5	4.22	4.3
1	1	1	2	3.5	3.44	3.6
1	1	2	1	3.25	3.89	4.8
1	1	1	1	6	4.11	5.3
1	1	1	2	7	5	4.37
1	1	1	2	7	6.67	5.6
1	1	1	1	7	4.33	5.26
1	1	1	1	6.75	4.67	4.4
2	2	1	1	4.75	6.33	6.4
1	1	1	1	7	5.67	5
1	1	1	2	7	5.67	5
2	2	1	2	6	3.78	2.8
1	1	1	2	6.75	5.44	6.2
1	1	1	2	6	4.11	5.4
2	2	1	2	6.25	5.22	5.1
2	2	1	2	5.5	4.33	4.4
2	2	1	2	4	4.11	4.3
1	1	1	3	4.25	3.89	5.1
1	1	1	2	5	5.33	5.8
1	1	1	3	4.5	5.22	5.6
2	2	1	3	6.5	5.11	4.2
2	2	2	2	5.5	6.33	5.3
1	1	2	2	7	6.67	5.5
1	1	1	2	7	6.44	5.5
2	2	1	3	4.75	4.33	5.8
1	1	1	2	4.25	4	3.7
2	2	1	2	4.75	4.67	6.7
1	1	1	2	5.5	4.56	3.6

Data generated or analyzed during this study

1	1	1	2	2.5	3.67	3.7
1	1	1	2	2.75	4.67	4
2	2	1	2	5.75	4.67	5.1
2	2	1	2	4	4.11	5.2
1	1	1	2	7	5	4.2
1	1	2	3	7	5.67	5.9
1	1	2	2	4	2.56	5
1	1	1	2	7	5.22	5.7
1	1	2	2	4.5	4.67	5.7
2	2	1	3	6.5	4.11	5
1	1	1	3	6.75	5.11	5.4
1	1	1	2	5.25	4.11	4.2
2	2	1	2	3.75	4.56	4.1
1	1	3	2	4	4	4.2
1	1	1	2	4.25	4	4
1	1	1	2	4.25	4.44	4.6
1	1	2	2	4.25	3.78	3.3
1	1	1	1	6.5	3.89	4.5
2	2	1	2	5.25	4.56	4.9
1	1	1	3	4.5	5.56	5.7
1	1	1	1	4.5	5.56	5.1
2	2	1	3	7	5.11	5.7
1	1	1	2	3.5	2.89	2.1
1	1	1	3	5	3.89	5.2
1	1	1	3	5.75	4.11	4.2
1	1	1	2	7	4.33	5.4
1	1	1	2	6.5	5	5.2
1	1	1	2	4.25	5	4.9
2	2	1	2	6	5.11	4.8
2	2	1	2	5	4.78	5
1	1	1	2	6	5.12	4.7
2	2	1	2	7	5.78	5.3
2	2	1	2	7	5.56	6.1
2	2	2	3	5.5	4.44	6.6
2	2	2	2	5.5	5.56	6.3
1	1	1	2	4.5	5.22	5.6
2	2	1	3	5.5	6.11	5.9
1	1	1	2	6	5.89	6.2
1	1	2	2	6	4.89	5.6
1	1	1	2	3.75	4	4.3
2	2	1	2	5.75	5	5
1	1	1	2	7	5.44	5.6
2	2	1	2	7	5.78	4.3
2	2	1	2	5.75	5.56	6.8
1	1	2	2	6.75	5.33	5.3
2	2	1	3	5.75	4	6.2
2	2	1	2	4.25	3.44	3
2	2	3	2	2.75	4.78	4.2
2	2	1	2	4.25	4	4
1	1	1	3	7	5	4.5
2	2	1	2	5.75	6.44	6
2	2	1	3	4	3.89	4.4
2	2	2	3	5	4.56	5.2

Data generated or analyzed during this study

1	1	2	2	5.75	4.11	5.4
1	1	2	2	5.5	5.11	2.8
1	1	1	2	7	5.56	5.2
2	2	1	3	5.75	3.78	6.2
2	2	1	2	5.75	3.78	6.2
1	1	1	2	4	4.56	3.3
1	1	1	3	6.5	4.34	6.2
1	1	1	2	5.5	5.67	6.7
2	2	1	2	6.75	5	5.63
1	1	1	3	6	4.11	5.4
